# Depression like-behavior and memory loss induced by methylglyoxal is associated with tryptophan depletion and oxidative stress: a new in vivo model of neurodegeneration

**DOI:** 10.1186/s40659-024-00572-4

**Published:** 2024-11-21

**Authors:** Md. Samsuzzaman, Seong-Min Hong, Jae Hyuk Lee, Hyunjun Park, Keun-A Chang, Hyun-Bum Kim, Myoung Gyu Park, Hyeyoon Eo, Myung Sook Oh, Sun Yeou Kim

**Affiliations:** 1 College of Pharmacy, Gachon University, #191, Hambakmoero, Yeonsu-gu, Incheon, 21936 Republic of Korea; 2 Department of Biochemistry and Molecular Biology, University of Maryland, Baltimore, MD 21201 USA; 3 Department of Health Sciences and Technology, Gachon Advanced Institute for Health Sciences and Technology (GAIHST), Gachon University, Incheon, Republic of Korea; 4 Neuroscience Research Institute, Gachon University, Incheon, Republic of Korea; 5 Department of Pharmacology, College of Medicine, Gachon University, Incheon, Republic of Korea; 6 Department of East-West Medical Science, Graduate School of East-West Medical, Kyung Hee University, Deogyeong-daero, Giheung-gu, Yongin-si, Gyeonggi-do 446-701 Korea; 7 MetaCen Therapeutics Company, Changnyong-daero 256 Beon-gil, Yeongtong-gu, Suwon-si, Gyeonggi-do 16229 Republic of Korea; 8 College of Pharmacy, Kyung Hee University, 26 Kyungheedae-ro Dongdaemun-gu, Seoul, 02447 Republic of Korea; 9 Gachon Institute of Pharmaceutical Science, Gachon University, #191, Hambakmoe-ro, Yeonsu-gu, Incheon, 21936 Republic of Korea

**Keywords:** Depression, Tryptophan, Methylglyoxal, BDNF, Tau, MAPK, Nrf-2/Ho-1/Trx

## Abstract

**Background:**

Depression and memory loss are prevalent neurodegenerative disorders, with diabetic patients facing an elevated risk of brain dysfunction. Methylglyoxal (MGO) formation, which is heightened in diabetes owing to hyperglycemia and gut dysbiosis, may serve as a critical link between diabetes and brain diseases. Despite the high prevalence of MGO, the precise mechanisms underlying MGO-induced depression and memory loss remain unclear.

**Results:**

We investigated the effect of MGO stress on depression like-behavior and memory loss to elucidate the potential interplay between MGO-induced tryptophan (Trp) metabolism impairment and oxidative stress in the brain. It demonstrates that MGO induces depression-like behavior in mice, as confirmed by the OFT, TST, FST, SPT, and EPM behavioral tests. MGO led to the depletion of Trp and related neurotransmitters as 5-HT, EPI, and DA in the mouse brain. Additionally, MGO reduced the cell count in the DG, CA1, and CA3 hippocampal regions and modulated TPH2 levels in the brain. Notably, co-treatment with MGO and Trp mirrored the effects observed after Trp-null treatment in neurons, including reduced TPH1 and TPH2 levels and inhibition of neuronal outgrowth. Furthermore, MGO significantly altered the expression of key proteins associated with neurodegeneration, such as p-Tau, p-GSK-3β, APP, oAβ, BDNF, NGF, and p-TrkB. Concurrently, MGO activated MAPKs through ROS induction, triggering a redox imbalance by downregulating Nrf-2, Ho-1, TXNRD1, Trx, Sirt-3, and Sirt-5 expression levels, NAD^+,^ and CAT activity in the mouse brain. This led to an accelerated neuroinflammatory response, as evidenced by increased expression of Iba-1, p-NF-κB, and the secretion of IL-6 and TNF-α. Importantly, Trp treatment ameliorated MGO-induced depression like-behavior and memory loss in mice and markedly mitigated increased expression of p-Tau, APP, p-ERK1/2, p-pJNK, and p-NF-κB in the brain. Likewise, Trp treatment also induced the expression of MGO detoxifying factors GLO-I and GLO-II and CAT activity, suggesting the induction of an antioxidant system and reduced inflammation by inhibiting IL-6 and TNF-α secretion.

**Conclusions:**

Our data revealed that MGO-induced depression like-behavior and memory deficits resulted from disturbances in Trp, 5-HT, BDNF, and NGF levels, increased p-Tau and APP expression, neuroinflammation, and impaired redox status (Nrf-2/Ho-1/TXNRD1/Sirt3/5) in the brain.

**Supplementary Information:**

The online version contains supplementary material available at 10.1186/s40659-024-00572-4.

## Background

Depression like-behavior and memory loss are the most common and recognizable cognitive function-impairing diseases that cause mortality and morbidity in South Asian and Western communities [1]. Diabetes mellitus (DM) is another cause of mortality and disease complications [2]. Studies on patients with depressive disorder and animals indicate possible underlying factors for this condition, such as impaired neuronal connectivity, decreased neuronal spine density, and hippocampal cell loss [3–6]. Several research groups have demonstrated a bidirectional relationship between DM and depression. Patients with DM are at a high risk of depressive disorders and memory loss [7, 8]. One statistical analysis found a correlation between depression, memory loss, and poorly controlled hyperglycemic conditions [9]. Hyperglycemia causes cognitive decline, neurodegeneration, dementia, and brain aging. The pathogenic mechanisms of brain damage due to hyperglycemia are too complex and involve a combination of oxidative stress, mitochondrial dysfunction, apoptosis, neuroinflammation, decrease in neurotrophic factors, changes in neurotransmitter level, and accumulation of amyloid β and tau phosphorylation [10–12]. Many studies have been conducted, but the results are inconsistent [13–17]. Previous studies have provided an ideal basis for the correlation between hyperglycemia and depressive disorders. In this context, hyperglycemia’s actual role and mechanism in brain diseases are critical issues.

Hyperglycemic conditions and gut bacteria are common sources of highly reactive metabolites. Among them, methylglyoxal (MGO) is the most reactive carbonyl species (RCS) [18]. MGO is detoxified into D-lactate *via *the glyoxalase system  *via* glutathione (GSH)-dependent pathway. MGO reacts with GSH to produce hemithioacetal, a substrate for GLO-I, which is further converted into S-D-lactoylglutathione by glyoxalase-I (GLO-I). Later, it is transformed into D-lactate through glyoxalase-II (GLO-II) to generate GSH [19]. Decreased levels or activity of GLO-I or GLO-II are a possible indicator of lower levels of GSH. Another mechanism of MGO reduction involves the nicotinamide adenine dinucleotide (NAD)-dependent dehydrogenase pathway. Hence, MGO is transformed to D-lactate using glycerol dehydrogenase and aldehyde dehydrogenase with the help of the cofactor NADH^+^ to NAD^+^ [20]. However, any disturbance in MGO detoxification leads to oxidative damage and changes in the redox status. Interestingly, MGO directly targets GSH or GLO and the thioredoxin (Trx) system to disturb redox homeostasis [21], and both systems use nicotinamide adenine dinucleotide phosphate hydrogen (NADPH)-provided reducing equivalents.

Oxidative stress is a biological process when normal antioxidant defense systems are disturbed [22]. It plays a critical role in diabetes-associated diseases and is also associated with depression. Neuronal and neurotransmitter loss in the brain is related to high levels of oxidative stress. Oxidative stress reduces antioxidant defense mechanisms such as nuclear factor erythroid-related factor (Nrf2), heme oxygenase (HO-1), superoxide dismutase (SOD), NAD^+^ activity, and catalase (CAT) enzyme activity. Oxidative stress activates nuclear factor kappa B (NF-κB) to enhance inflammation in the brain [23]. Therefore, a proper balance between oxidative stress and defense systems is urgently needed. Thus, clinical investigations suggest that high levels of MGO in the serum are closely associated with rapid cognitive decline in elderly patients [24, 25]. Additionally, it has been demonstrated that the extrinsic oral administration of MGO to cells or rats induces behavioral or physiological changes [26]. Despite these interesting findings, the mechanisms of MGO-induced depressive disorder and memory loss remain unclear.

The brain-derived neurotrophic factor (BDNF)/tropomyosin receptor kinase B (TrkB) and phosphoinositide 3-kinase (PI3K)- protein kinase B (Akt) signaling pathways maintain long-term potential (LTP) in the hippocampus, synaptic plasticity, and neuronal differentiation and survival. In addition, they also prevent Tau protein phosphorylation and amyloid precursor protein (APP) accumulation in the brain [27–30]; those factors are closely linked to depression and memory impairment. Moreover, BDNF protects hippocampal neurons from oxidative damage by activating the Nrf2 and HO-1 signaling pathways [31]. Nevertheless, disruption or dysregulation of BDNF/TrkB signaling impairs learning and memory and induces depression.

Tryptophan (Trp), a common substance used by the gut microbiota, is an essential amino acid, and one of the most important roles of Trp is to act as a precursor for neurotransmitters such as serotonin (5-HT), which has a strong role in mood, stress response, appetite, and especially in depression [32]. There are two pathways for Trp metabolism. Trp is converted to neurotransmitters by tryptophan hydroxylase 2 (TPH-2), and the second one is indoleamine 2,3-dioxygenase (IDO), where the end product is quinolinic acid, which triggers toxicity inside the cell. Clinical studies revealed that patients with lower levels of Trp showed depressive and mood-changing characteristics, although the role of Trp in depression like-behavior remains controversial, as reported by research groups [33].

The gut-brain axis is now a topic of research interest that represents the relationship between the brain and gut, including the autonomic and enteric nervous systems and the gastrointestinal microenvironment. However, any disturbance in gut homeostasis networks in the intestine may lead to severe conditions, affecting brain mood and behavioral development [34]. Nevertheless, MGO is a major bacterial product of the anaerobic glycolysis of carbohydrates in the large intestine. However, the actual mechanisms of gut-derived MGO in the intestine during depression and memory function remain largely unknown.

In this study, we aimed to understand whether MGO, administered rectally, can induce depression like-behavior and memory loss in mice and to determine whether impaired BDNF/TrkB signaling, and antioxidant defense system or activation of oxidative stress are involved in the underlying effects of MGO. Therefore, we investigated the impact of MGO stress on depression like-behavior and memory loss to elucidate the potential interplay between MGO-induced Trp metabolism impairment and oxidative stress in the brain.

## Materials and methods

### Material and chemicals

4-(2-hydroxyethyl)-1-piperazineethanesulfonic acid (HEPES, H4034), L-glutamine (G-8540) and D-glucose (G-7528) were purchased from Sigma (St. Louis, MO, USA). Minimum essential medium (MEM, LM 007 − 01) and Hank’s balanced salt solution (HBSS, LB 003 − 01) were bought from Welgene (Kyungsan, South Korea). Donor horse serum, heat inactivated (HS, S0900-500) was obtained from Biowest (Nuaillé, France). Penicillin streptomycin (LS0202-02) was acquired from Gibco BRL (Rockville, MD, USA). Phosphorylated (p)-tropomyosin receptor kinase (Trk) A (cat. #9141), TrKA/B (cat. #4619), extracellular signal-regulated kinase (ERK1/2, cat. #9102), p-ERK1/2 (cat. #9101), c-Jun N-terminal kinases (JNK, cat. #9252), p-JNK (cat. #9251), p38 (cat. #9212), p-p38 (cat. #9101), HO-1 (cat. #70081), p-Akt (cat. #9271), Akt (cat. #9272), p-glycogen synthase kinase-3β (p-GSK-3β, cat. #9336), GSK-3β (cat. #9315), p-IkBα (cat. #2859), IkBα (cat. #4812), p-NF-κB (cat. #3033), NF-κB (cat. #4764) were purchased from Cell Signaling Technology (Danvers, MA, USA). Antibodies against glyoxalase 1 (GLO-I, cat. sc0133144), glyoxalase 2 (GLO-II, cat. sc271786), receptor for advanced glycation end products (RAGE, cat. sc365154), glyceraldehyde-3-phosphate dehydrogenase, (GAPDH, cat. sc32233), silent mating type information regulation 2 homologue (Sirt)-1 (cat. sc135792), Sirt-2 (cat. sc28298), Sirt-3 (cat. sc365175), Sirt-4 (cat. sc135797), Sirt-5 (cat. sc271635), and Sirt-7 (cat. sc365344) were purchased from Santa Cruz Biotechnology (Santa Cruz, CA, USA). p-TrkB (cat. ab229908), p-Tau (cat. ab32057), Tau (cat. ab254256), TPH1 (cat. ab52954), TPH2 (cat. ab288067), nerve growth factor (NGF, cat. ab66459), thioredoxin reductases1 (TXNRD1, cat. ab124954), Trx (cat. ab273877), glucocorticoid receptor (GR, cat. ab183127), and ionized calcium binding adaptor molecule-1 (Iba-1, cat. ab16588) antibodies were purchased from Abcam (Cambridge, UK). BDNF (cat. A11028) antibody was received from Abclonal. APP (cat. 14974982) and amyloid β-protein (Aβ) oligomers (oAβ, cat. AHB0052) were obtained from Invitrogen (MA, USA).

### Animals and study design

**Fig. 1:**
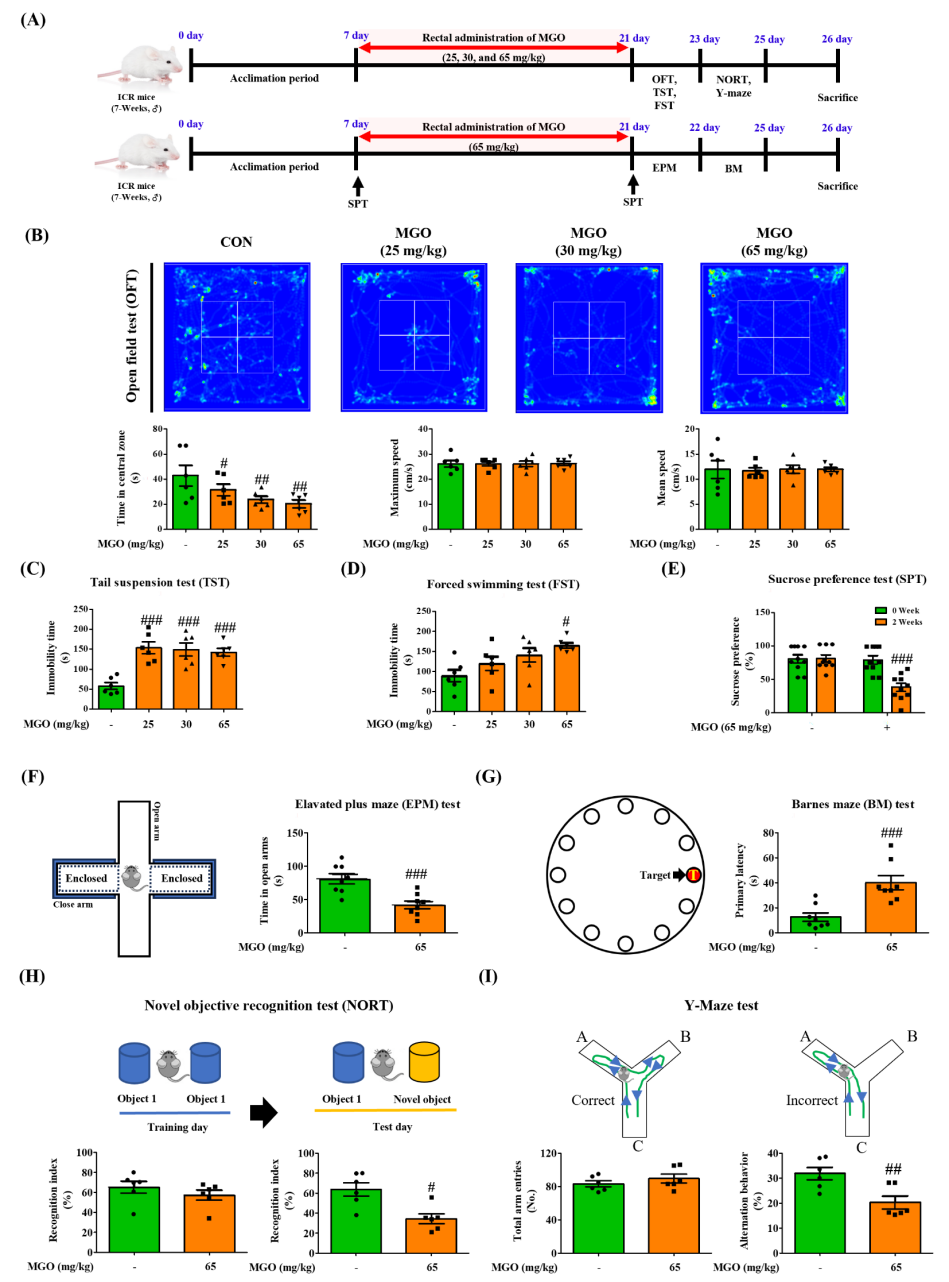
Depression-like behavior and working memory loss in mice. (**A**) Schematic diagram of the experimental plan. (**B**) The total distance, time spent and speed in the open field test (OFT). (**C**) The immobility time in the tail suspension test (TST) chamber (**D**) The immobility time in the forced swimming test (FST) chamber (**E**) The percentage of sucrose consumption in the sucrose preference test (SPT). (**F**) The time spend in the opened arms from elevated plus maze (EPM) test (**H**) The latency to reach the target in the barnes maze (BM) test after training 3 days. (**H**) The percentage of recognition index in novel object tool in the novel objective recognition test (NORT). (**I**) The number of total arm entries and the summary of the percentage of alteration in Y-maze test. All results obtain from behavior tests was calculated using SMART3.0 SUPER PACK. The values are presented as mean ± SEM (n = 6–8). ^#^*p* < 0.05, ^##^*p* < 0.01, and ^###^*p* < 0.001 vs. control group (CON)

ICR mice (7-weeks-old, male) were acquired from Orient Bio (Gyeonggi-do, Korea) and placed in a standard temperature-(22 ± 2 °C) with 65% humidity and a 12 h/12 h light/dark cycle. The Animal Care Committee of the Center of Animal Care and Use at Gachon University (GIACUC-R2020008) reviewed and approved all experimental guidelines. One week after the acclimation period, the ICR mice were divided into five groups: control (CON, n = 8), 25 mg/kg of MGO (n = 8), 30 mg/kg of MGO (n = 8), 65 mg/kg of MGO (n = 6–8), and 65 mg/kg of MGO + 40 mg/kg of Trp (MGO + Trp, n = 8). Trp (40 mg/kg) was oral administered to experimental ICR mice for 2 or 3 weeks. MGO (25, 30, or 65 mg/kg) and Trp (40 mg/kg) were administered to experimental ICR mice for 2 or 3 weeks *via* rectal injection [30% v/v glycerol in phosphate-buffered saline (PBS, pH 7.4)] (see Figs. 1A and 8A, and 9A).

### Open field test (OFT)

The OFT was conducted in an open plastic box (45 cm × 45 cm × 45 cm), as reported by Ueno et al. with modifications [35]. The box zone was divided into 24 grids of 11.25 × 11.25 cm. The mice were individually placed in the center of an open plastic box and allowed to observe freely, after which a video was recorded for 5 min. An open plastic box was cleaned with 70% ethanol. The total distance, time spent, and speed in the central zone(s) was analyzed using SMART3.0 SUPER PACK (Panlab; Harvard Apparatus, Barcelona, Spain).

### Tail suspension test (TST)

TST was performed as mentioned by Ueno et al. with a bit of change [35]. The TST was conducted in a TST chamber (60 cm length, 60 cm height, 11.5 cm depth, and 15 cm width), and the mice were suspended using painless tape-based fixation. Before recording, all the mice were adapted to the TST chamber for 2 min. The mice were individually placed in the TST chamber, and videos were recorded for 4 min. Next, the immobility of the mice was analyzed using the SMART3.0 SUPER PACK.

### Forced swimming test (FST)

The FST was performed as reported by Kang et al. with slight modifications [35]. It was performed in an FST chamber (50 cm height × 20 cm diameter) filled with water (at a level of 30 cm) set at room temperature (25 ± 1 °C). Before recording, all mice were pre-adapted to the FST chamber for 2 min and then placed in the FST chamber, after which the video was recorded for 4 min. The immobility of the mice was evaluated using the SMART3.0 SUPER PACK.

### Sucrose preference test (SPT)

SPT was performed as reported by Luo et al. with slight modifications [36]. The mice were adapted to 2% sucrose solution (w/v) were placed in each cage for 2 days, and then deprived of water and food for 16 h, followed by SPT. After 48 h, two bottles with 2% sucrose solution (20 ml) and tap water (20 ml) were freely given. Three hours later, sucrose and water consumption (ml) were recorded. The percentage of sucrose preference was calculated as sucrose consumption / (sucrose consumption + water consumption).

### Elevated plus maze (EPM) test

The EPM test was analyzed as described in previous report [37]. The comprised of open arms (35 cm × 5 cm) and closed arms (35 cm × 5 cm × 15 cm) that crossed from a central area (5 cm × 5 cm). The floor and walls were painted black, the entire maze was elevated to a height of 45 cm above floor. The mice were individually placed in the center of EPM tool and allowed to observe freely, after which a video was recorded for 5 min. The total distance and time spent in open arms were analyzed using SMART3.0 SUPER PACK.

### Barnes maze (BM) test

The BM test was constructed from black polyethylene and consist of circular are (45 cm diameter) with 12 circular holes (4.5 cm diameter) as see in Fig. 1G. The maze was elevated 50 cm above the floor. The escape box (35 cm × 25 cm × 15 cm) was constructed black polyethylene. Mice were placed circular central zone and then trained to reach the escape box for 4 days. The mean latency time to reach the target was analyzed using SMART3.0 SUPER PACK.

### Novel objective recognition test (NORT)

Multiple experiments began with an acquisition trial designed to acquire mice with two similar objects. This was followed by a probe trial in which a familiar object was substituted with a novel object to assess recognition memory. After each trial, the box and objects were thoroughly cleaned to eliminate olfactory cues from preceding mice. Plastic toys of various shapes were used as objects in this examination. Object exploration was defined as beginning to sniff the object from a maximum distance of 1 cm or less, excluding climbing and sitting on the object from exploratory activities. Recognition memory was gauged by comparing the time spent exploring the novel object to the time spent exploring the familiar object. The training trial lasted 5 min, after which the mice were returned to their home cages at 10-minute intervals. Subsequently, the probe trial, which lasted for 3 min, involved replacing one familiar object with a novel one, and the time spent exploring each object was recorded.

### Y-Maze test

The mice were positioned at the terminus of one arm in a symmetric Y-maze featuring arms 40 cm in length, 8 cm in width, and 20 cm in height. The mice were allowed to explore the apparatus freely for 5 min. An overhead camera captured the sequence of arm entries (e.g., ACBCABCBCA). An alteration was registered when the mouse accessed three different arms within a triad of overlapping triplet sets (e.g., in the ACBCABCBCA sequence, five alternations were tallied). Spontaneous alternation was determined by counting the instances when the mouse sequentially entered each of the three arms of the maze, divided by the maximum potential alternations. The maximum number of alternations was calculated as the total number of arm entries minus two.

### Measurement of Trytophan (Trp), 5-hydroxytryptophan (5-HTP), and serotonin (5-HT) levels in the plasma

Trp, 5-HTP, and 5-HT levels were analyzed as described by Fuertig et al. [38] with slight modifications. Briefly, mouse plasma was mixed with 0.1% formic acid and acetonitrile (ACN), vortexed, and mixed within 30 s for 5 min. The reaction samples were centrifuged, vortexed with 0.1% formic acid, and injected into the Agilent LC 1100 series LC-MS/MS system (CA, USA). Chromatography was performed using an Agilent ZORBAX Extend-C18 column (1.0 × 150 mm, 3.5 μm) operated at 30 °C. The solvent system consisted of mobile phase A [100% ACN)] and B (0.1% formic acid in distilled water) at a ratio of 1:1 (v/v, %). To detect tryptophan, 5-HTP, and serotonin in the analytes, the MS/MS system was operated in positive (ESI+), negative (ESI-), and multiple reaction monitoring modes.

### Measurement of neurotransmitter levels in brain tissues

The dopamine (DA), epinephrine (EPI), and 5-HT levels were analyzed using the protocol reported by Planchez et al. [39]. After the sacrifice of mice, the whole brain was removed and homogenized in 0.1 M perchloric acid (PCA) (10 mg/µL). The samples were then centrifuged at 12,000 rpm for 30 min. The supernatant was filtered using 0.2 μm filters and injected into the HPLC system (Waters Corp., MA, USA). The samples (20 µL) were analyzed using a Kromasil C18 column (150 mm × 4.6 mm, 5 μm) with an electrochemical detector. The mobile phase consisted of 2% citric acid, 2% K_2_HPO_4_, 1 mM EDTA, 1.2% MeOH, and 7 mg/mL sodium octyl sulfate. The detector conditions were + 0.008 V and a 0-100 nA sensitivity range. The flow rate was maintained at 1.0 ml/min.

### Measurement of Trp levels in cell medium and extracts

Trp levels were analyzed according to a previously reported method [40]. N2a cells were seeded in a 60 Φ dish, washed with cold PBS, centrifuged, and extracted with ice-cold 50% methanol/50% piperazine –N (PIPES)-EDTA at -20 °C for 5 to 10 min. After the cell extracts were separated by centrifugation, the samples were injected into the HPLC system. The samples were analyzed using a Sepax HP-C18 column (250 mm × 4.6 mm, 5 μm) with 15 mM potassium phosphate and 2.7% ACN as an isocratic mobile phase at a detection wavelength of 220 nm. The flow rate was 0.8 mL/min, and the column temperature was maintained at 30 °C.

### Neurite outgrowth assay

N2a cells were seeded into a 6-well plate (4.0 × 10^5^ cells/well) and incubated for 4 h. After incubation, the cells were treated with or without MGO (500 µM) and tryptophan-free medium for 48 h. IncuCyte^®^ ZOOM Imaging System was used to evaluate the neurite outgrowth, neurite length, and cell morphology of treated or untreated cells.

### Field excitatory postsynaptic potential (fEPSP) activity

fEPSP activity was performed as described in previous report [41]. The prepared the organotypic hippocampal tissue on micro-electrode array was tested as shown below. Bipolar electrical stimulation was applied to stratum radiatum region of CA (Cornu Ammonis) 2 and CA3 for inducing the Schaffer collateral and commissural pathways. The intensity of bipolar stimulation was set at 80 µA, 120 µs per phase which was optimized level to offer 40–65% of the maximum hippocampal tissue response. Theta-burst stimulation (TBS) consisted of 300 biphasic pulses, 3 trains at 100 Hz for 1 s (train interval for 5 min). Each experiment for inducing LTP was planned with total 100 min protocol. There were 40 min of test recording of field excitatory postsynaptic potential (fEPSP) at one stimulation per minute (baseline; first 10 min), 10 min of TBS, and 50 min of post-TBS fEPSP measurement. The point for inducing Schaffer collateral and commissural pathways was chosen by morphological structure of hippocampal tissue and appropriate response by bipolar electrical stimulation. While the experiments were processing, the slices were continuously perfused with fresh aCSF at the rate of 3 ml/min. MGO and CNQX were treated alone or together with aCSF 10 min after recording. Unrefined analog MEA signal and acquired fEPSPs level from triggering amplitudes over 40 µV were digitized by MC Rack from Multi Channel Systems. And customized MATLAB (v.7.0.1, Mathworks. Inc.) was used for removing stimulus artifacts and integrating the evoked field potential trajectory.

### Primary hippocampal neuron culture

Timed pregnant (TP) 17 days (SD) rats were purchased from Koatech (Korea). The hippocampal tissue was enzymatically dissociated using 0.25% trypsin solution (Gibco). The dissociated hippocampal tissue was washed with 1x HBSS (Gibco) and resuspended in a neurobasal medium containing 2% B27, 2-mM L-glutamine, and 1% penicillin-streptomycin (Gibco). Hippocampal neurons were seeded in 96-well plates or 18-mm coverslips pre-coated with 0.1 mg/ml poly-L lysine (Sigma). Hippocampal neurons were plated in 96-well plates at a density of 2 × 10^4^ cells/well or on 18-mm coverslips at a density of 3 × 10^4^ cells/coverslip, respectively. Half of the culture medium was replaced every 3–4 days. Primary hippocampal neurons were maintained in a 5% CO^2^ humidified incubator at 37 ℃ for 14 days. At DIV 11–13, primary hippocampal neuron media was changed with or without tryptophan before treatment with 500 µM of MGO. WST-1 (Roche, Basel, Switzerland) was used to measure the metabolic activity of viable cells. WST-1 levels were determined according to the manufacturer’s instructions. After incubation with MGO for 24 h, WST-1 reagent was added to the wells. The primary hippocampal neurons were incubated at 37 ℃ in 5% CO_2_ for 2 h. The absorbance of the reaction medium was measured at 450 nm using a Vector X4 multilabel plate reader (Perkin Elmer).

### Spine density analysis

Hippocampal neurons were fixed in 4% paraformaldehyde (PFA) solution for 20 min and washed with 1x PBS. The fixed hippocampal neurons were permeabilized in PBS containing 0.1% Triton X-100 for 15 min and blocked with 1% bovine serum albumin (BSA) for 45 min. The hippocampal neurons were stained with primary antibodies for 1 h, followed by incubation with fluorescence-conjugated secondary antibodies or phalloidin 488 (Thermo Fisher Scientific) for 2 h. The hippocampal neurons were washed with PBS and mounted on glass slides. Images of the hippocampal neurons were captured using a Zeiss LSM 700 confocal microscope with a 20x objective. Hippocampal neurons were fixed in a 4% PFA solution for 20 min at room temperature and washed using 1x PBS. Fixed hippocampal neurons were permeabilized in PBS containing 0.1% Triton X-100 for 15 min and blocked 1% BSA for 45 min at room temperature. Hippocampal neurons were stained with primary antibody for 1 h at room temperature followed by microtubule-associated protein 2 (MAP2; 1:200, mouse-IgG; Sigma-Aldrich). Secondary antibodies were goat anti-mouse 594 (1:200; Invitrogen, UK) and cytopainter phalloidin 488 (1:200; Invitrogen, UK), and incubated for 2 h at room temperature. The hippocampal neurons were washed with PBS and mounted on the slide glass. Images of hippocampal neurons were captured by a Zeiss LSM 700 confocal microscope with a 20x objective. The 20x images were taken with 1024 × 1024 pixels. Dendritic spines were analyzed with Zen 2.3 SP1 software. Z-stack images were collected at 20 μm intervals and then compressed into a 2D image with maximal intensity projection.

### Cytokines levels, NAD^+^ and catalase (CAT) activity measurement

The cytokines including interleukin (IL)-6, -10, and tumor necrosis factor-α (TNF-α) from mouse plasma were analyzed using enzyme-linked immunosorbent assay (ELISA) kits (R&D system, Minneapolis, MN, USA). For measuring the NAD^+^ or CAT activity, the mouse brain tissue was homogenized in buffer, and then quantified using a colorimetric or quantification BioVision assay kit according to the manufacturer’s instructions.

### Western blot analysis

N2a cells and brain tissues lysed in PRO-PREP™ protein extraction solution (iNtRON, Seoul, Korea) at -20 °C for 24 h. After the cell lysates were separated by centrifugation, protein concentration was determined using the Bradford assay. Proteins (30–50 µg) were separated using sodium dodecyl sulfate-polyacrylamide gel electrophoresis and transferred to polyvinylidene fluoride (PVDF) membranes using a Trans-Blot^®^ Turbo™ blotting system. The membranes were then blocked using 5% skim milk for 1 h and incubated overnight at 4 °C with primary antibodies. After overnight incubation, the membranes were washed and incubated with secondary antibodies at room temperature (25 °C) for 1 h. The bands were detected using a ChemiDoc™ XRS + imaging system (Bio-Rad, CA, USA).

### Quantitative real-time PCR

Total RNA was extracted from cultured N2a cells using TRIzol^®^ reagent (Invitrogen, CA, USA). A total of 1 µg of RNA was reverse transcribed to complementary DNA using the PrimeScript™ RT reagent Kit (Takara Bio, Otsu, Japan), according to the manufacturer’s protocol. Quantitative polymerase chain reaction (qPCR) was performed using SYBR^®^ Premix Ex Taq™ (Takara Bio) on an Mx3005P qPCR System (Agilent Technologies). The Supplementary Material lists the primer sequences (Supplementary Table S1). Glyceraldehyde 3-phosphate dehydrogenase (GAPDH) served as a housekeeping gene.

### Immunochemistry (IHC) analysis

IHC assay was performed in described as described in previous report [42]. The mice were anaesthetized and then perfused with cold saline. The brain in 4% PFA was fixed for 1 day at 4 °C and incubated in 30% sucrose solution for 3 days at 4 °C. The coronal Sect. (25 μm) were taken by using cryostat (Cryotome, Thermo Electron Corporation) and stored at 4 °C. Each section washed with 0.2% Triton X-100 in PBS and then incubated in blocking solution including 0.5% BSA and 3% goat serum in 0.4% PBS with Tween 20). The prepared sections were incubated with primary antibody (1: 300; cat. NBP2-16908, Novus Biological, CO, USA) overnight at 4 °C. Afterward, the sections were washed and then AlexaFluor 555 Donkey anti-rabbit IgG secondary antibody (1: 500; A-31572, Invitrogen, MA, USA) was incubated for 1 h at room temperature. The stained sections were mounted onto slides using Fluoromount™ aqueous mounting medium (Sigma-Aldrich) with DAPI Vector Laboratories, CA, USA). The stained slides were observed under a laser scanning confocal microscope (Nikon A1+) and analyzed using the NIS-Elements imaging software. Iba-1stained brain sections were analyzed by region of interest (ROI) intensity ratio (%), and the microscopy magnification set as 4× – 10×.

### Histological analysis

Paraffin blocks of large intestine, small intestine, and brain tissues were fixed in 4% PFA. After fixation, all tissues were dehydrated by exposure to ethanol, washed with xylene, embedded in paraffin blocks, and sectioned to 5 μm thicknesses. Sections were stained using a hematoxylin and eosin (H&E) staining kit (Sigma-Aldrich). For immunohistochemical analysis, sections were incubated in 0.1% protease K in phosphate-buffered saline (PBS) for antigen retrieval and incubated in 3% H_2_O_2_ in PBS for 15 min. Sections were incubated with 1% normal horse serum in phosphate-buffered saline (PBS) for 20 min. After blocking, the sections were incubated with GR (1: 100, Abcam, UK), TPH1 (1: 100, Abcam, UK) and TPH2 (1: 100, Abcam, UK) over night in a shaker at 4 °C. The sections were washed and incubated with biotinylated respective IgG secondary antibodies at room temperature (25 °C) for 1 h. The sections were then rinsed with PBS and incubated with VECTASTAIN^®^ ABC reagent for 30 min. The antibody expression was detected using 3,3-diaminobenzidine (DAB). The stained tissue slides were observed and photographed using a Nikon Eclipse 80i microscope (Nikon, Tokyo, Japan) at 10× magnification, and detected intensity was calculated using the ImageJ software (NIH, Bethesda, MD).

### Molecular docking

For protein preparation, the 3D crystal structure of sirt-3 (4V1C) and sirt-5 (5XHS) were retrieved from the protein data bank and prepared by removing water molecules and ligands. To prepare the ligand for docking, the 3D chemical structure of MGO was acquired from PubChem. The MGO was then uploaded to Discovery Studio, and the structure was modified by adding hydrogen and cleaning the geometry. The prepared proteins and ligands were uploaded to AutoDock Vina. Then, hydrogen was added, and the torsion parameter was set following grid box settings at 126, 126, and 126 Å for X, Y, and Z, respectively. The final output files from AutoGrid were saved in the PDBQT format. The obtained PDBQT file was used for docking analysis. A Lamarckian genetic algorithm (LGA) was selected as the best conformer. All other parameters needed for the docking analysis were used at default settings in AutoDock. Docking visualization between the protein and ligand was performed using the Discovery Studio Visualizer.

### Statistical analysis

All data are presented as the mean ± standard error of the mean. All statistical analyses were performed using Prism 5.0 (GraphPad Software Inc., CA, USA), SPSS software (version 25.0; IBM SPSS Statistics Inc., IL, USA), and t-tests. Statistical comparisons between control and experimental groups were performed using Bonferroni’s test for multiple comparisons and one-way analysis of variance. Tukey’s post hoc comparison tests after one-way ANOVA were used to calculate the statistical significance of the cell viability assay and spine density analysis. Differences were considered statistically significant at *p* < 0.05.

## Results

### Methylglyoxal enhanced depression-like behavior and memory loss in ICR mice

To clarify whether MGO affects locomotion, cognition, and anxiety-like behavior in ICR mice, we performed several depressive behavioral tests. The results of the open field test (OFT), the most extensively used method to assess anxiety behavior, revealed that the MGO (25, 30, and 65 mg/kg)-treated groups displayed a significant reduction in the time spent in the central zone compared to the control (CON) group (Fig. 1B). Less time spent by the mice in the central area indicated fearful characteristics, as it has already been established that depressed subjects do not spend more time in the central zone [35]. And the maximum and mean speed of each group was not significant different. The tail suspension test (TST), another technique to assess depression-like activity in ICR mice, confirmed that MGO (25, 30, and 65 mg/kg) induced longer immobility time in the mice compared with the CON group (Fig. 1C). Similarly, we found that MGO (30 and 65 mg/kg) triggered immobility in ICR mice in the forced swimming test (FST) (Fig. 1D). We further studied the potential role of MGO in depression *via* SPT and EPM test (Fig. 1E and F). Notably, MGO (65 mg/kg) dramatically decreased sucrose consumption in comparison with CON group. Besides, our designed mice model showed that the spent time in open arm significantly decreased in the EPM test. Therefore, the behavioral test data suggested depression like-behavior- and anxiety-inducing abilities of MGO in mice. The BM, NORT and Y-maze tests were used to examine the effects of MGO on memory function in mice. In the BM test, MGO group significantly increased the latency time and distance to reach the target area (Fig. 1G), indicating that MGO treated mice have an impairment in hippocampus or loss of motivation. During the training phase, no significant differences were observed in the exploration times for the two objects between the CON and MGO-treated groups. During the test, the percentage of NORT was significantly lower in the vehicle treated with MGO group than in the vehicle treated with 30% glycerol-PBS (Fig. 1H). The Y-maze test was used to analyze the effects of MGO on working memory. The number of arm entries and the proportion of arm entry triads in which the mouse visited every arm without repetition were recorded. As shown in Fig. 1I, MGO treatment did not change the percentage of arm entries compared with the CON group, but the alteration percentage was significantly reduced (Fig. 1I). These results indicated the ability of MGO to induce memory deficits.

### Methylglyoxal promoted neuronal loss and mitigated long-term potentiation (LTP) in the hippocampus area

**Fig. 2:**
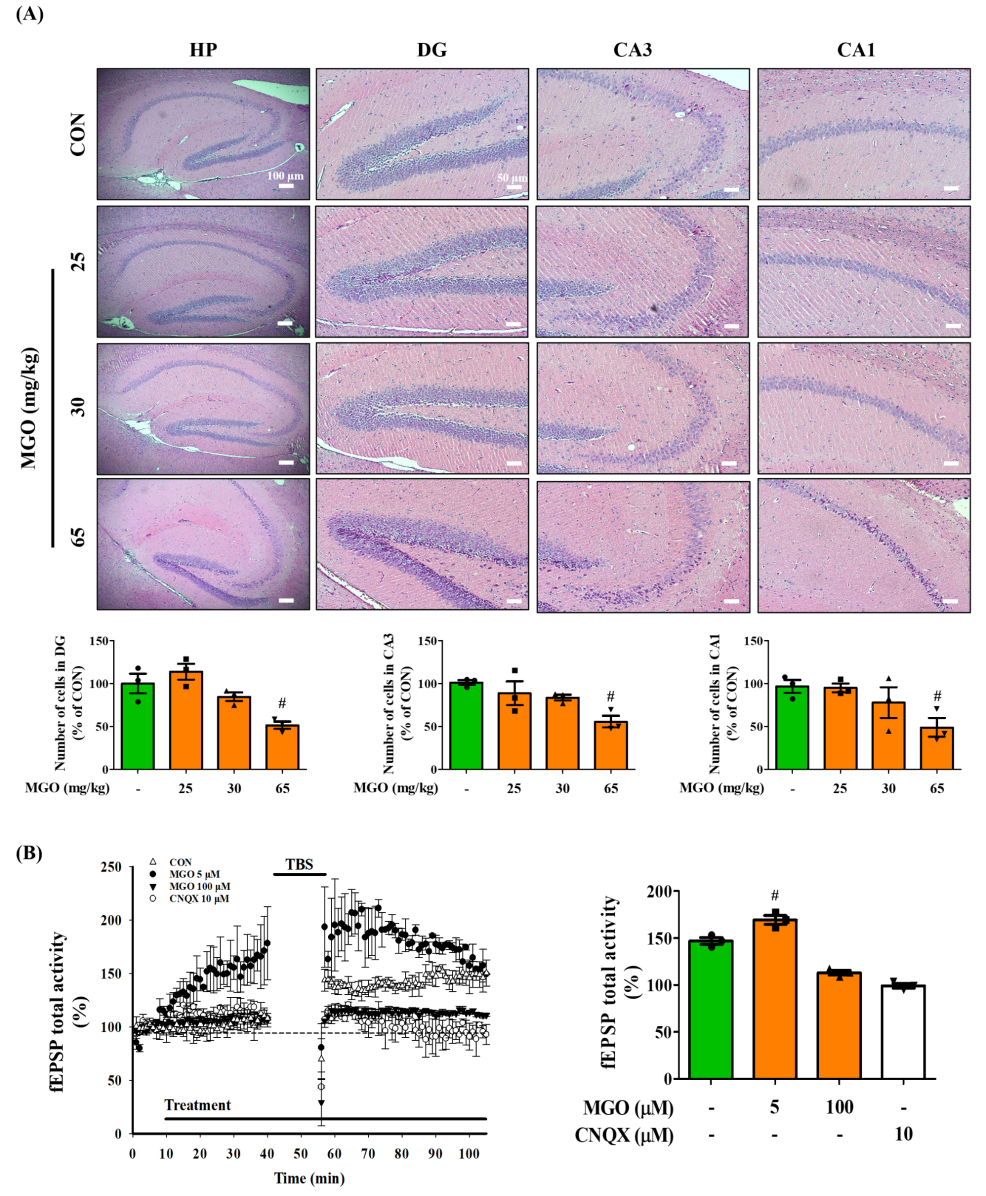
Methylglyoxal (MGO) facilitated hippocampal cell loss and lowered the long-term potential (LTP) activity. (**A**) Histological staining of brain sections in dentate gyrus (DG), cornu ammonis 3 (CA3), and cornu ammonis 1 (CA1) regions of hippocampus (HP). Scale bar: 50–100 μm. The number of cells in the DG, CA3, and CA1 regions from selected fields per image. The values were calculated using ImageJ software. The values presented as mean ± SEM (n = 3). (**B**) LTP from all recordings in the CON, MGO-, and CNQX-treated hippocampus. The fEPSP total activity from 30 to 40 min after TBS in the CON, MGO-, and CNQX-treated hippocampus (n = 3). Control, treated with nothing after 10 min of the baseline; MGO, treated with 5 and 100 µM of MGO after 10 min of the baseline; CNQX, treated with 10 µM of CNQX after 10 min of the baseline. ^#^*p* < 0.01 vs. control group (CON)

A reduced number of cells and structural changes in the hippocampus are vitally connected to major depressive disorder, learning, and memory functions [43]. Hence, we postulated that MGO may harm neuronal cells in different parts of the hippocampus (HP). Therefore, we investigated the effects of MGO treatment on the dentate gyrus (DG), cornu ammonis 3 (CA3), and cornu ammonis 1 (CA1) of the mouse hippocampus. Neurons in the pyramidal and granular cell layers of the CA3, CA1, and DG were counted. Interestingly, MGO significantly lowered the number of neuronal cells in the DG, CA3, and CA1 regions of the hippocampus at a dose of 65 mg/kg (Fig. 2A) compared to the CON group, suggesting that MGO induced neuronal cell death in the hippocampus of mice. We also investigated the effect of different concentrations of MGO (5 and 100 µM) on hippocampal LTP. The time-dependent changes in field excitatory post-synaptic potential (fEPSP) activity and the mean % of fEPSP from 30 to 40 min after TBS were pooled for analysis. Treatment of MGO at 5 µM increased post-TBS-stimulated fEPSP (169.38 ± 8.03%) compared to the CON group (146.96 ± 6.36%). However, a relatively high concentration of MGO (100 µM) inhibited the induction of LTP by TBS (113.14 ± 4.33%) (Fig. 2B). Like the effects seen upon treatment with 100 µM MGO, induction of LTP was completely blocked upon treatment with cyanquixaline (CNQX) (99.18 ± 4.47%), an AMPA receptor antagonist, with no significant difference compared with 100 µM MGO treatment (Fig. 2B). Therefore, our results suggest that MGO blocks LTP in the hippocampus.

### Shared targets of methyglyoxal and depression like-behavior

In total, 171 possible targets of MGO were identified by obtaining the Pathway Assembly from the Literature Mining-an Information Search Tool (PALM-IST) database and validating it in the PubMed database. In addition, 14,839 genes related to depression were retrieved from GenBank. Based on the intersection of prospective targets of depression and MGO, 161 potential genes were identified (Supplementary Fig. S1A). Gene ontology (GO) biological and KEGG pathway analyses were performed to understand the underlying mechanisms of the genes involved in depression. The primary terms in the GO analysis were modulation of synaptic transmission, synaptic signaling, behavioral changes, and cell-cell signaling (Supplementary Fig. S1B). Additionally, the KEGG pathway was mainly involved in neuroactive signaling, CAMP signaling, neurodegenerative pathway activation, PI3K-Akt signaling, and MAPK signaling pathways (Supplementary Fig. S1C). Moreover, hub gene prediction analysis identified BDNF, IL-6, and IL-1β as the hub genes (Supplementary Fig. S1D). These results suggest the importance of these genes in neurotransmitter regulation, synaptic signaling, BDNF, PI3K-Akt, MAPK, and inflammatory signaling in depression like-behaviour.

### Methylglyoxal depleted tryptophan in the plasma and hippocampus of ICR mice

**Fig. 3:**
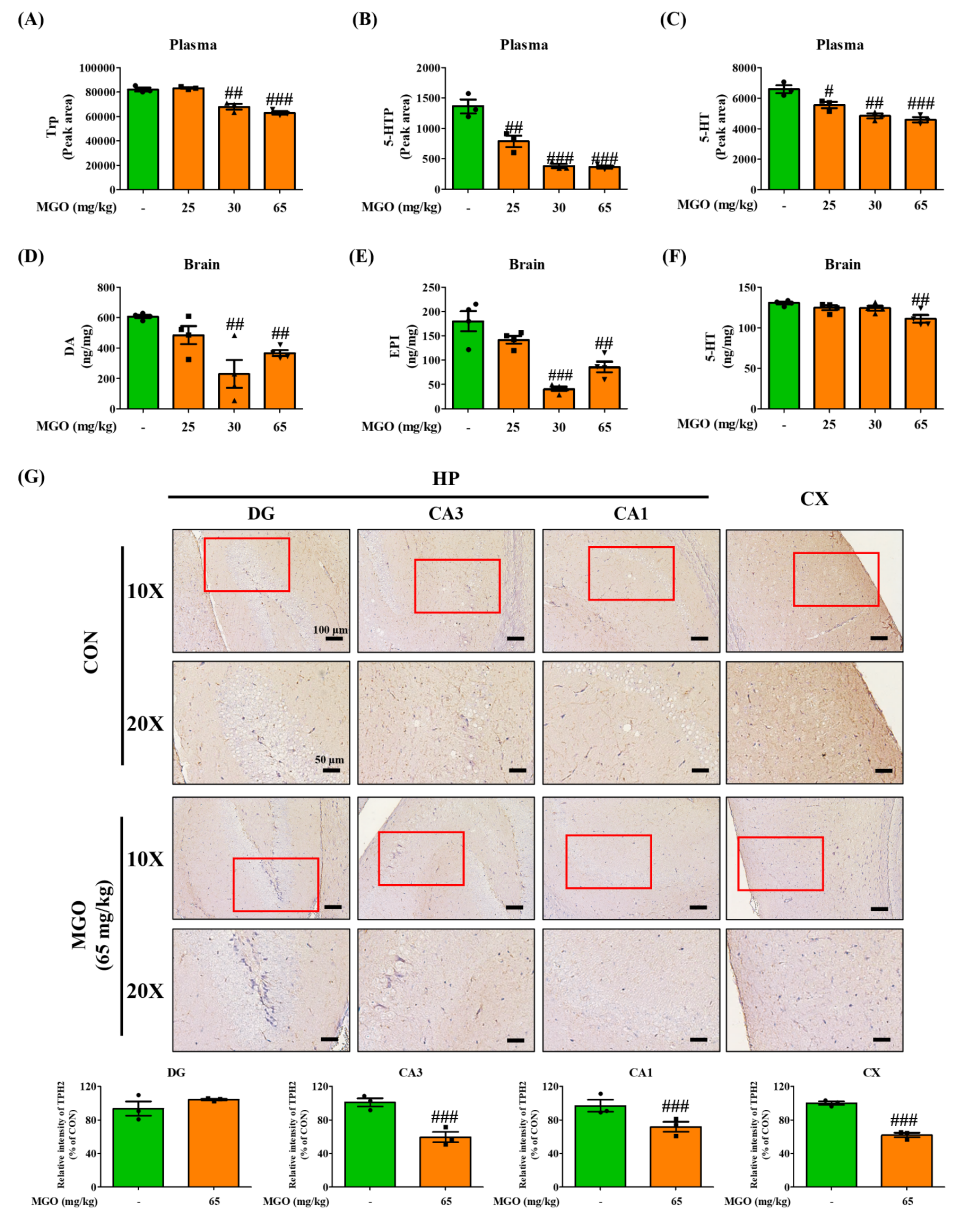
Methylglyoxal (MGO) suppressed tryptophan (Trp) and neurotransmitter levels in the plasma and brain of mice. (**A**–**C**) Levels of Trp, 5-hydroxytryptophan (5-HTP), and serotonin (5-HT) in the mice plasma. (**D**–**F**) The levels of dopamine (DA), epinephrine (EPI), and serotonin (5-HT) in the mice whole brain. (**G**) The TPH2 levels in the DG, CA3, CA1) and cortex (CX) regions by IHC staining assay. Scale bar: 100 μm (20× magnification). The TPH2 intensity were conducted by calculating the expression in the DG, CA3, CA1, and CX from selected fields per image. The values were calculated using ImageJ software. All data presented as mean ± SEM (n = 3). ^#^*p* < 0.05, ^##^*p* < 0.01, and ^###^*p* < 0.001 vs. control group (CON). HP, hippocampus

Trp, a neurotransmitter precursor, is known to accelerate depression [44], which led us to hypothesize that MGO-induced depression in mice may be mediated by modulating the levels of Trp and related factors such as 5-HTP, 5-HT, TPH1, and TPH2. Therefore, we examined the effects of MGO on these parameters using LC-MS/MS system and IHC staining assay. Mouse plasma was subjected to LC-MS/MS analysis for Trp, 5-HTP, and 5-HT. We found that MGO markedly downregulated tryptophan levels at doses of 30 and 65 mg/kg and those of 5-HTP and 5-HT in a dose-dependent manner in mouse plasma (Fig. 3A – C). Furthermore, decreased DA, EPI, and 5-HT levels were observed in the brain tissue at 65 mg/kg of MGO (Fig. 3D – F). As TPH-2 is mainly expressed in the brain, we inspected TPH2 expression in different sections of the hippocampus because lower levels of tryptophan have also been associated with impaired hippocampal function. As illustrated in Fig. 3G, while MGO lowered TPH2 expression in the CA3, CA1, and cortex (CX) sections at a dose of 65 mg/kg compared to the CON group, it did not affect TPH2 expression in the DG section (Fig. 3G). TPH1 expression levels were detected in cortical brain sections *via* immunostaining, as the mood may depend on an integral pathway linked to the cortex. Anatomically, the cortex is directly connected to the brain’s hippocampus. Our experimental results showed that MGO also notably reduced TPH1 expression in the cortex, even at a dose of 25 mg/kg (Supplementary Fig. S2). However, TPH1 expressed in the intestine provides serotonin to the brain. Its levels also declined in the intestines of mice treated with MGO at 65 mg/kg (Supplementary Fig. S3), with reduced colon length. These data indicate that the MGO-induced depressive effects in mice may be due to an impaired tryptophan metabolic pathway in the brain and intestine.

### Tryptophan deficiency linked to MGO-induced brain dysfunction

**Fig. 4:**
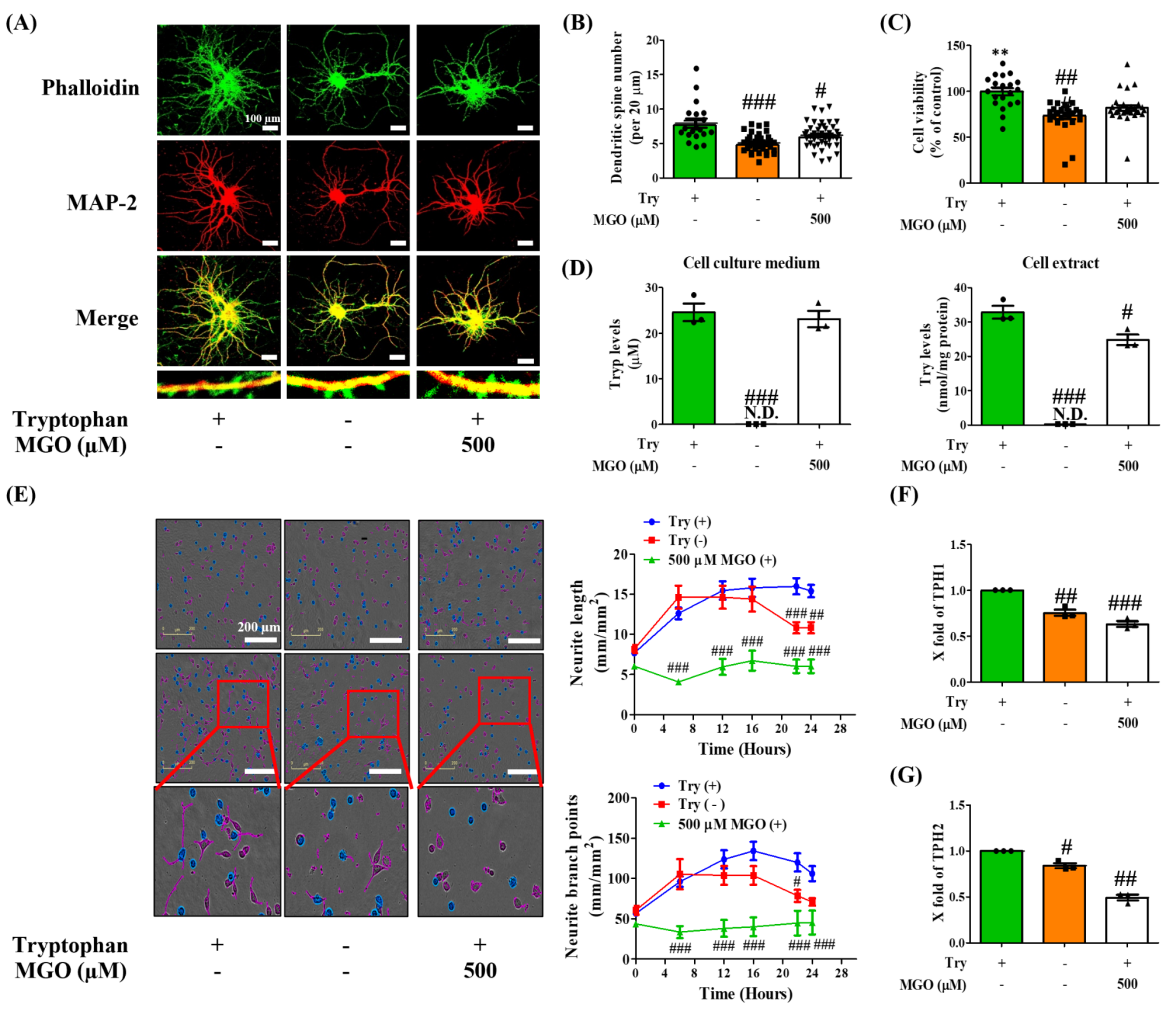
Methylglyoxal (MGO) induced neuronal cell loss upon tryptophan depletion. (**A**, **B**) Immunofluorescent staining of primary hippocampal neurons. The cells were treated with MGO (500 µM) and Trp-(-) or Trp (+) medium for 24 h. Dendritic spine density was counted from neuronal dendritic segments. Scale bar: 100 μm (20× magnification). (**C**) The cell viability of primary hippocampal neurons. (**D**) The levels of Trp in cell culture medium and cell extracts by using HPLC system. (**E**) The neurite length images of N2a cells by treating MGO (500 µM) and Trp-(-) or Trp (+) medium for 24 h. Quantitative analyses of neurite outgrowth were conducted by calculating the number of neurite lengths from randomly selected fields per well. Scale bar: 200 μm (10× magnification). (**F**, **G**) The mRNA expression levels of TPH1, and TPH-2 in N2a cells. All data presented as mean ± SEM (n = 3). ^#^*p* < 0.05, ^##^*p* < 0.01, and ^###^*p* < 0.001 vs. Trp (+) medium treatment

To confirm the Trp depletion role in MGO-induced depression and memory dysfunction, the dendritic spine density of the primary hippocampal neuron in MGO-treated (500 µM) or untreated [Trp (+)] and Trp-null-treated [Trp (-)] group was compared. Surprisingly, the spine density of neuronal cells was significantly lower in the Trp (-)-treated group than in the Trp (+)-treated control group. Similarly, MGO treatment reduced spine density compared with the Trp(+) group, even after treatment with Trp (Fig. 4A, B). Moreover, MGO- and Trp (-)-induced toxicity was observed in primary hippocampal neuronal cells (Fig. 4C). To verify MGO’s role in Trp depletion, we quantified Trp levels in the presence of MGO. Interestingly, the Trp levels were markedly reduced in the MGO-exposed group between cell culture medium and cell extract (Fig. 4D). We used the same experimental plan to evaluate neurite outgrowth in another neuronal (N2a) cell type. We found that the introduction of MGO or tryptophan-null environment to neuronal cells strongly shortened the neurite length and branch point compared to the Trp (+)-treated control group (Fig. 4E). Additionally, TPH1 and TPH2 levels were downregulated in N2a cells (Fig. 4F, G). These results demonstrate that MGO causes Trp depletion, which is one of the factors responsible for depression like-behavior and memory impairment.

### Methylgloxal modulated phosphorylation of Tau (Ser396), Akt (Ser473)/GSK-3β (Ser9) and affected BDNF/NGF axis in mice an impairment in the hippocampus

**Fig. 5:**
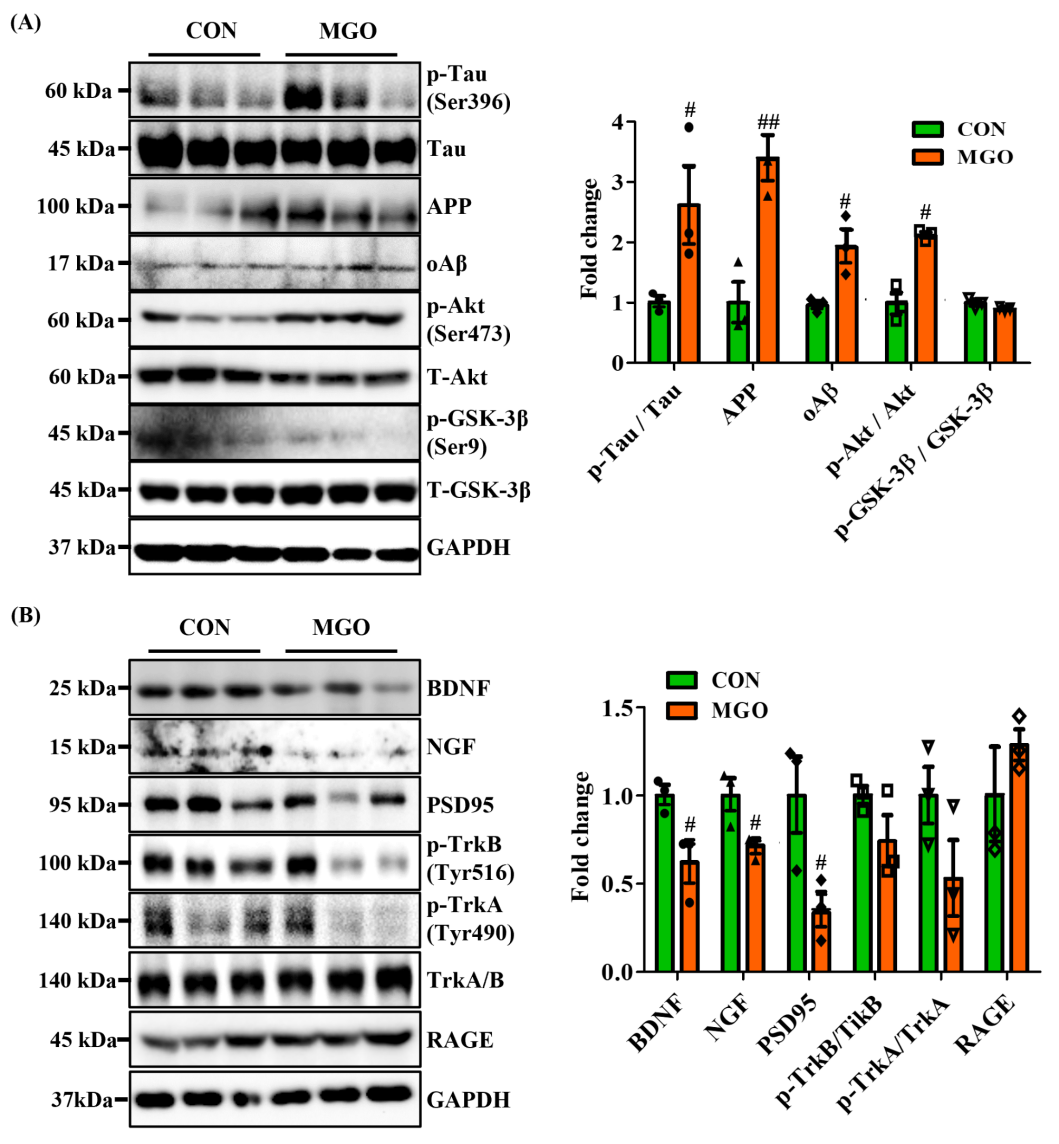
Methylglyoxal (MGO) activated Tau and Akt/GSK-3β phosphorylation and reduced BDNF/TrkB or NGF/TrkB protein expression in mice. (**A**) The expressed levels of p-Tau, Tau, APP, oAβ, p-Akt, Akt, p-GSK-3β, GSK-3β, and GAPDH by western blotting assay. (**B**) The expressed levels of BDNF, NGF, PSD95, p-TrkB, TrkB, p-TrkA, TrkA, RAGE, and GAPDH by western blotting assay. The densitometry graph of each band. All data presented as mean ± SEM (n = 3). ^#^*p* < 0.05 and ^##^*p* < 0.01 vs. control group (CON). CON, control group. MGO, 65 mg/kg of MGO rectal injection group

To explore the molecular mechanism of MGO-induced depression like-behavior and memory deficit, the phosphorylated form of Tau (Ser396), Akt (Ser473), GSK-3β (Ser9), and expression levels of APP and oAβ were examined. Tau phosphorylation and oAβ deposition have been associated with rapid cognitive decline, and the Akt/ GSK-3β (Ser9) signaling axis acts as an upstream regulator [45]. Western blotting results exhibited, compared to the control, that MGO dramatically increased the phosphorylation of Tau and Akt but reduced the phosphorylated form of GSK-3β (Ser9). MGO did not show any significant effects on the total form status of Tau (Ser396), Akt (Ser473), and GSK-3β (Ser9) (Fig. 5A). Likewise, MGO considerably increased the expression of oAβ and APP in mice as compared to CON group (Fig. 5A). However, we next addressed whether activation of the BDNF/TrkB or NGF/TrkA signaling axis leads to the phosphorylation of Tau or Akt kinases in mice because of their critical role in depression like-behavior and memory decline. Our data revealed that MGO significantly suppressed BDNF, NGF, and PSD95 protein expression compared to the CON group. PSD95 is a postsynaptic density protein that regulates the trafficking and localization of synaptic receptors, thus contributing to synaptic development and plasticity [46, 47]. Similarly, MGO sharply diminished the phosphorylation of TrkB (Tyr516) and TrkA (Tyr490) in mice compared with the control panel, whereas the total form remained unchanged. Further, we assessed RAGE protein expression, as it works as a receptor for MGO-derived advanced glycation end products (AGEs) to activate downstream MAPK, Akt, and GSK-3β signaling axis. In contrast, RAGE expression levels did not change in the MGO-exposed mice (Fig. 5B). The above data prove that MGO-induced brain dysfunction through the phosphorylation of Tau (Ser396), increased expression, or deposition of APP, and oAβ in mice, and the activation of the Akt (Ser473) or reduction of GSK-3β (Ser9) signaling axis was independent of BDNF/TrkB or NGF/TrkA signaling.

### Methylglyoxal-activated the expression of NF-κB to facilitate neuroinflammation

**Fig. 6:**
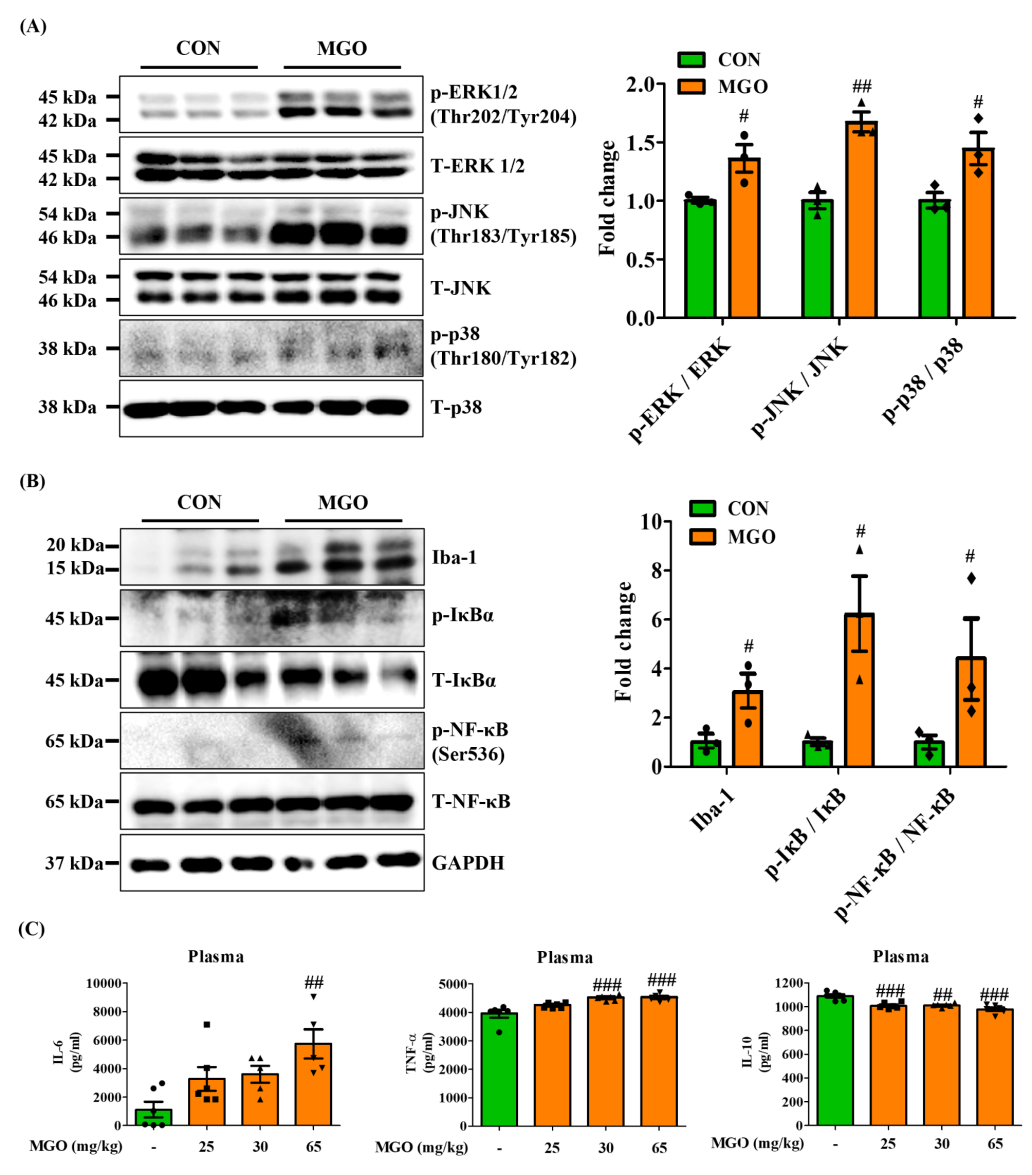
Methyglyoxal (MGO) facilitated MAPK activation and enhanced inflammation in mice. (**A**, **B**) The expressed levels of p-ERK1/2, ERK1/2, p-JNK, JNK, p-p38, p38, Iba-1, p-IκB, IκB, p-NF-κB, NF-κB, and GAPDH by western blotting assay. Densitometry graph of each band. (**C**) The secreted levels of IL-6, TNF-α, and IL-10 cytokines in the plasma by ELISA assay kit. All data presented as mean ± SEM (n = 3–6). ^#^*p* < 0.05, ^##^*p* < 0.01, and ^###^*p* < 0.001 vs. control group (CON). CON, control group. MGO, 65 mg/kg of MGO rectal injection group

MAPK signaling plays a crucial role in Tau phosphorylation, and the activation of NF-kB subunits impacts the expression of pro-inflammatory cytokines [47]. Therefore, we investigated the active form expression of MAPK and NF-κB in brain tissue. Western blot data indicated that 65 mg/kg MGO caused a dramatic elevation in the phosphorylation of ERK1/2 (Thr202/Tyr204), JNK (Thr183/Tyr185), and p-38 (Thr180/Tyr182) compared with control mouse brain tissue (Fig. 6A). MAPK, especially JNK, is known to activate NF-κB under oxidative stress. The data showed that MGO significantly upregulated phosphorylation of NF-κB (Ser536) levels in the brain tissue (Fig. 6B). Next, we examined the levels of proinflammatory cytokines, as NF-κB is an upstream regulator of inflammatory cytokines. MGO significantly raised pro-inflammatory cytokines, IL-6, and TNF-α secretion and strongly reduced anti-inflammatory IL-10 cytokine secretion (Fig. 6C). Furthermore, Iba-1expression level, an activated microglial cell marker [48], was examined under MGO stress. As expected, Iba-1 protein expression was notably higher in MGO-treated mice than in mice treated with the control vehicle (PBS) (Fig. 6B). These findings indicated that MGO-induced MAPK activation might be involved with the Tau phosphorylation. Thus, it also activates NF-κB in the brain to induce neuroinflammation.

### Methylglyoxal downregulated the expression of antioxidant defense system

**Fig. 7:**
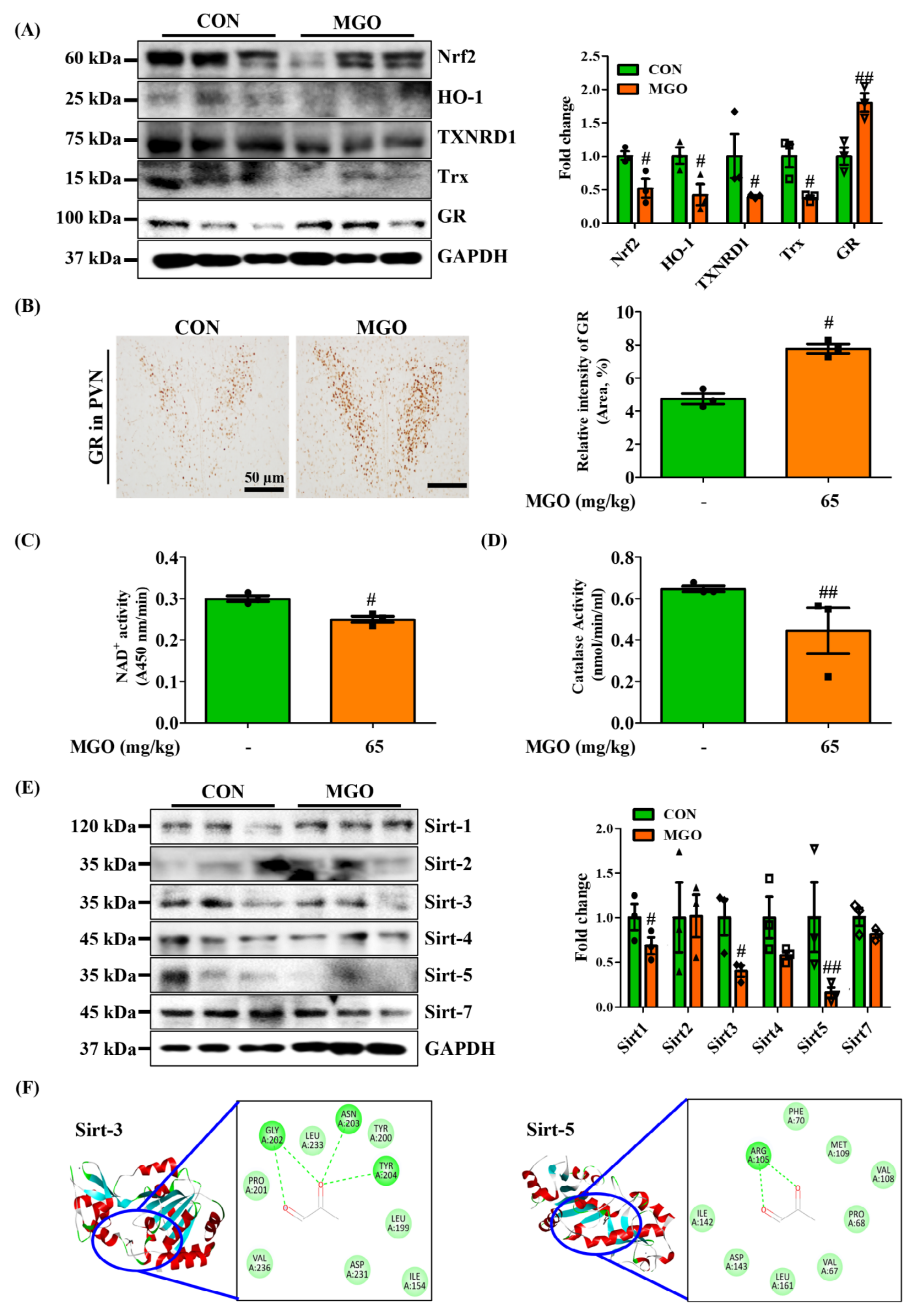
Methylglyoxal (MGO) down-regulated Nrf-2/Ho-1, Trx, and Sirt family proteins expression and upregulated GR expression in mice. (**A**) The expressed levels of Nrf-2, HO-1, Trx, TXNRD1, GR, and GAPDH by western blotting assay. The densitometry graph of each band. (**B**) Immunostaining detection of GR. The densitometry graph of each figure. Scale bar: 50 μm (20× magnification). (**C**, **D**) NAD^+^ and CAT activity by colorimetric assay kit. (**E**) The expressed levels of Sirt-1, Sirt-2, Sirt-3, Sirt-4, Sirt-5, Sirt-7, and GAPDH by western blotting assay. (**F**) Molecular docking analysis between MGO and Sirt-3/Sirt-5 using AutoDock Vina software. All data presented as mean ± SEM (n = 3). ^#^*p* < 0.05 and ^##^*p* < 0.01, and ^###^*p* < 0.001 vs. control group (CON). CON, control group. MGO, 65 mg/kg of MGO rectal injection group

We studied the oxidative status of mouse brains to determine how MAPK is activated in the presence of MGO without the BDNF or NGF signaling axis. MAPK proteins, such as ERK1/2, JNK, and p-38, are prime responders to oxidative stress, and the antioxidant defense mechanism is disrupted during stress [49]. We first analyzed antioxidant mechanism-related factors, such as Nrf2, HO-1, TXNRD1, Trx, and GR. We determined that 65 mg/kg MGO significantly downregulated Nrf2, HO-1, TXNRD1, and Trx expression levels in mice compared with the CON group (Fig. 7A). In contrast, MGO triggered GR expression levels compared with CON group, which is known to induce oxidative stress. We also confirmed the high expression of GR in MGO-treated mice through immunostaining (Fig. 7B). In addition, MGO markedly reduced NAD^+^ levels (Fig. 7C), as it is an important component of the MGO detoxification process [50], and GSH for anti-oxidant activity. Moreover, MGO moderately inhibited CAT activity (Fig. 7D). To confirm our findings, we examined the effects of MGO on sirtuin family proteins. Sirtuins (Sirt) play a regulatory role in balancing antioxidant and redox status [51]. Western blotting data revealed that MGO strongly suppressed Sirt-3 and Sirt-5 protein expression in the brain compared to the CON group (Fig. 7E). In addition, MGO showed higher binding affinity for Sirt-3 and Sirt-5 proteins, as confirmed by molecular docking analysis. MGO displayed a binding score of about − 4.31 and − 4.81 Kcal/mol to Sirt-3 and Sirt-5, respectively. MGO formed three strong hydrogen bonds by interacting with the GLY202, ASN203, and TYR204 residue of Sirt-3 and one strong hydrogen bond with the ARG105 residue of Sirt-5 (Fig. 7F). However, changes in the Sirt-1, Sirt-2, Sirt-4, and Sirt-7 expression levels were not significant in the presence of MGO in mice. The above data demonstrate that MAPK was activated through oxidative stress induced by MGO, which might play a critical role in Tau phosphorylation.

### Tryptophan mitigated methylglyoxal-induced depression and memory dysfunction by inhibiting Tau phosphorylation, oxidative stress, and inflammation in mice

**Fig. 8:**
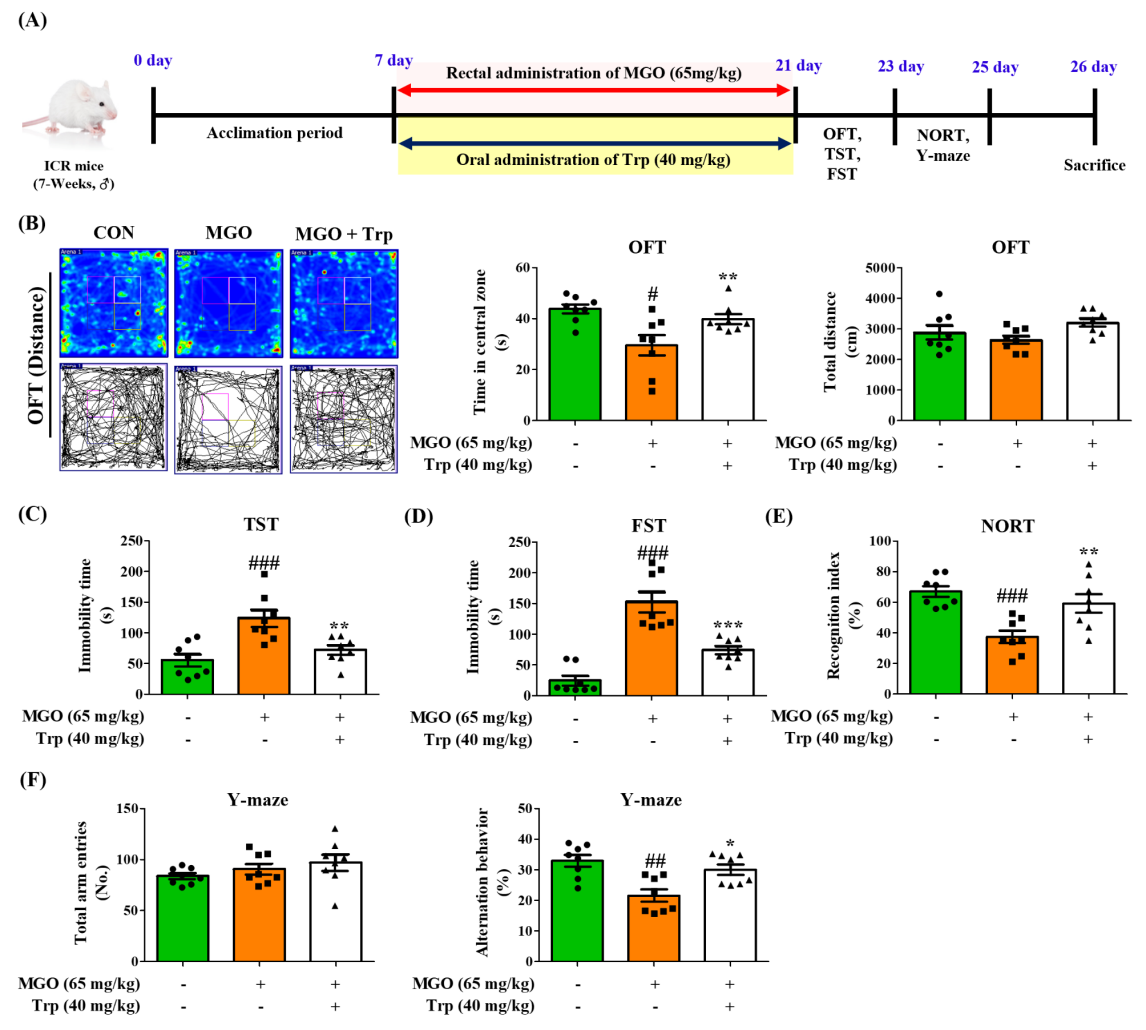
Tryptophan (Trp) rescued methylglyoxal (MGO) induced depression and memory dysfunction in mice. (**A**) Schematic diagram of the experimental plan. (**B**) The total distance, time spent and speed in the open field test (OFT). (**C**) The immobility time in the tail suspension test (TST) chamber (**D**) The immobility time in the forced swim test (FST) chamber. (**E**) The percentage of recognition index in novel object tool in the novel objective recognition test (NORT). (**F**) The number of total arm entries and the summary of the percentage of alteration in the Y-maze test. All results obtain from behavior tests was calculated using SMART3.0 SUPER PACK. The values are presented as mean ± SEM (n = 8). ^#^*p* < 0.05, ^##^*p* < 0.01, and ^###^*p* < 0.001 vs. control group (CON). ^*^p < 0.05, ^**^p < 0.01, and ^***^p < 0.001 vs. MGO-induced group (MGO). CON, control group. MGO, 65 mg/kg of MGO rectal injection group. MGO + Trp, 65 mg/kg of MGO rectal injection with 40 mg/kg of Trp oral administration group

**Fig. 9:**
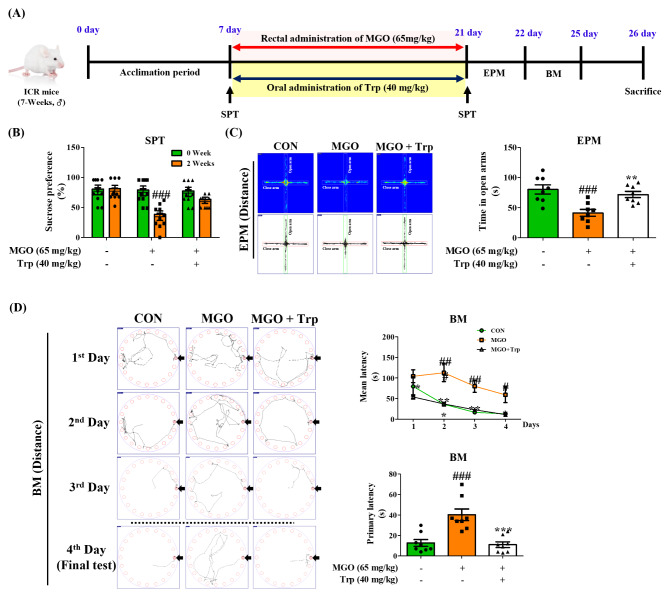
Tryptophan (Trp) recovered methylglyoxal (MGO) induced depression and memory loss behavior in mice. (**A**) Schematic diagram of the experimental plan. (**B**) The percentage of sucrose consumption in the sucrose preference test (SPT). (**C**) The total distance and time spend in the opened arms from elevated plus maze (EPM) test. (**D**) The total distance and latency to reach the target in the barnes maze (BM) test for 4 days. The mean latency time to reach the target for 4 days were analyzed. All results obtain from behavior tests was calculated using SMART3.0 SUPER PACK. The values are presented as mean ± SEM (n = 8). ^###^*p* < 0.001 vs. control group (CON). ^**^p < 0.01 and ^***^p < 0.001 vs. MGO-induced group (MGO). CON, control group. MGO, 65 mg/kg of MGO rectal injection group. MGO + Trp, 65 mg/kg of MGO rectal injection with 40 mg/kg of Trp oral administration group

To clarify whether MGO-induced depression like-behavior and memory loss were due to Trp deficits, oxidative stress-activated MAPK signaling, or neuroinflammation, we exogenously administered Trp at a concentration of 40 mg/kg by rectal injection into ICR mice in the presence or absence of MGO (Figs. 8A and 9A). Surprisingly, Trp treatment significantly reversed the time spent in the central zone in the OFT, which was reduced by MGO treatment (Fig. 8B). In the TST and FST, the immobility time was prolonged in the MGO treatment mice compared to the CON group, but the immobility time was reduced in Trp-treated mice (Fig. 8C and D). We also examined the effects of Trp in mice using NORT and Y-MAZE analyses in the presence or absence of MGO. Trp rescued the ability of mice to recognize new objects, which was reduced by MGO (Fig. 8E). The alteration ability of mice was also tested. Interestingly, the alteration percentage of mice treated with Trp was significantly higher than that of MGO-treated mice (Fig. 8F). The total number of arm entries did not change in the CON, MGO-, or Trp-treated mice (Fig. 8F).

**Fig. 10:**
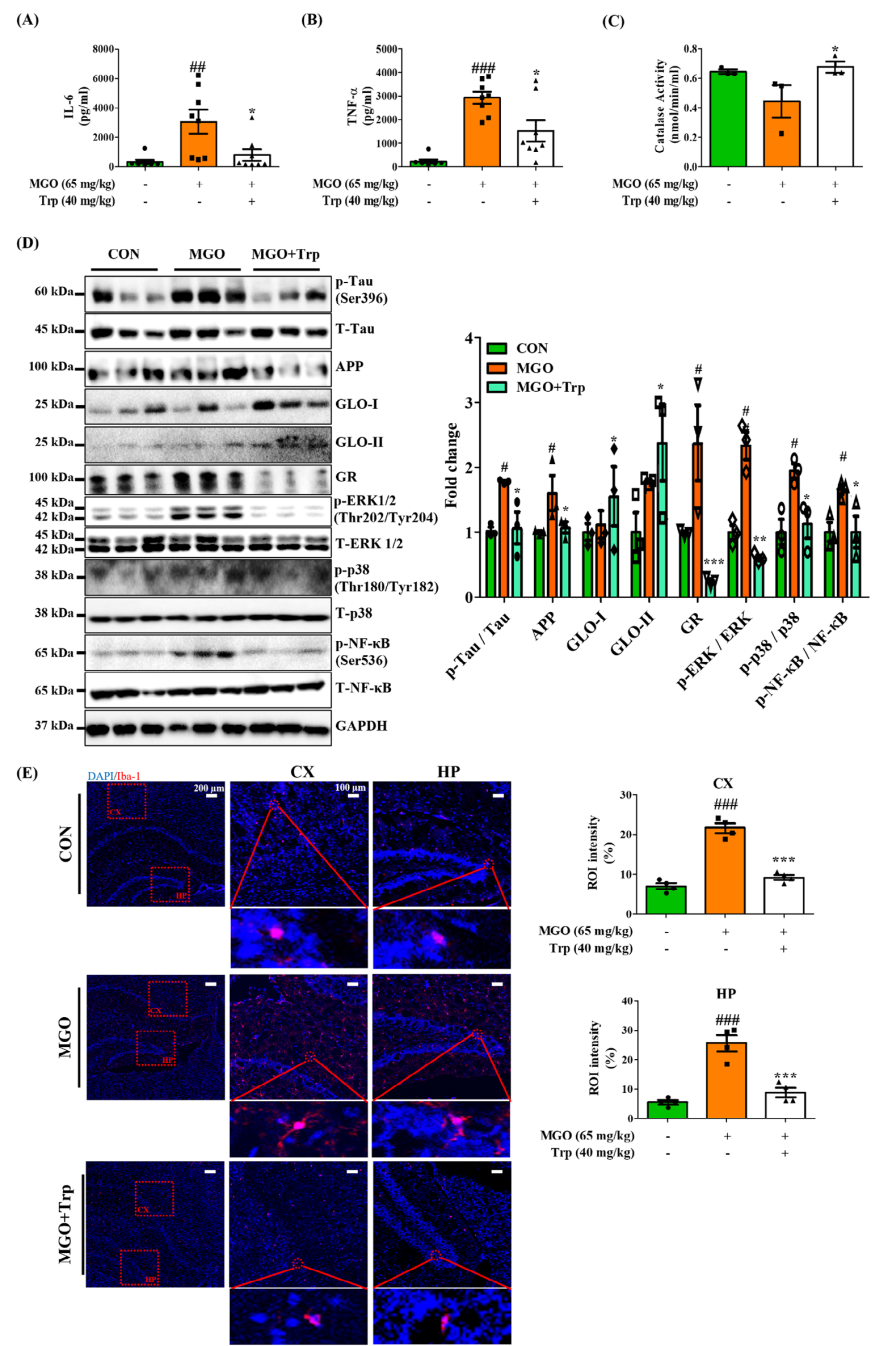
Tryptophan (Trp) mitigated methylglyoxal (MGO)-induced depression and memory loss by regulating the AD and HPA axis, glyoxalase system, oxidative stress, and inflammation markers in mice (**A**, **B**) The secreted levels of IL-6 and TNF-α by ELISA assay kit. (**C**) CAT activity by colorimetric assay kit. (**D**) The expressed levels of p-Tau, Tau, APP, GLO-I, GLO-II, GR, p-ERK1/2, ERK1/2, p-p38, p38, p-NF-kB, NF-kB, and GAPDH by western blotting assay. (**E**) Representative Iba-1 immunofluorescent red signal in brain. Scale bar: 100 μm (10× magnification) – 200 μm (4× magnification). The activated microglia in hippocampus (HP) and cortex (CX) was represented as a region of interest (ROI) intensity ratio (%). The values are presented as mean ± SEM (n = 3). ^##^*p* < 0.01 and ^###^*p* < 0.001 vs. control group (CON). ^*^p < 0.05, ^**^p < 0.01 and ^***^p < 0.001 vs. MGO-induced group (MGO). CON, control group. MGO, 65 mg/kg of MGO rectal injection group. MGO + Trp, 65 mg/kg of MGO rectal injection with 40 mg/kg of Trp oral administration group

We further examined the several behavior models of the anxiety- and memory loss such as SPT, EPM, and BM test. A shown in Fig. 9B, Trp-treated mice was significantly improved the sucrose consumption in comparison with MGO group in SPT. In the EPM test, the Trp-treated mice dramatically increased the spent time and total distance (Fig. 9C). In addition, Trp reduced primary latency to reach the target area in the BM test (Fig. 9D). These results suggested that Trp have a strong a strong ameliorative effect on MGO for promoting the depression and memory loss. Moreover, we evaluated Trp treatment effects in MGO-induced neuroinflammation, and the results were noticeable. Trp reduced pro-inflammatory cytokines IL-6 and TNF-α secretion in the plasma as compared to the MGO-treated group (Fig. 10A, B), but increased the CAT activity compared to those in the control, MGO, and mice (Fig. 10C).

To elucidate the molecular mechanism underlying the effects of Trp on MGO in mice, we investigated the role of Trp on MGO-activated Tau phosphorylation, MAPK signaling, oxidative stress, and neuroinflammation. Analysis results proved that Trp significantly diminished the MGO-induced phosphorylation of Tau, APP, and oAβ expression levels (Fig. 10D). We assessed the effects of Trp on oxidative stress-induced MAPK activation, GLO-I, GLO-II, GR expression, and catalase activity in MGO-treated mice. Trp suppressed MGO-induced phosphorylation of ERK1/2 and p-38 in brain tissue. Trp significantly increased GLO-I and GLO-II protein expression levels in MGO-exposed depressed mice, suggesting its MGO-scavenging efficacy. Moreover, the brain of Trp-treated mice sharply decreased GR expression levels compared those in the MGO-treated mice group (Fig. 10D). Further, we evaluated Trp treatment effects in MGO-induced neuroinflammation related transcription factors, and Trp exclusively reduced NF-κB phosphorylation as compared to the MGO-receiving mice group (Fig. 10D).

Finally, we postulated to do IHC staining for Iba-1 expression in mice brain, specifically hippocampus and cortex. Surprisingly, we found that MGO significantly increased the number of Iba-1 positive brain cells in hippocampus and cortex, whereas Trp dramatically reduced in MGO-induced mice (Fig. 10E). These results suggest that Trp protects mice from MGO-induced depression and memory loss through its MGO scavenging capacity, regulation of oxidative stress homeostasis, and reduction of inflammation in the brain.

## Discussion

Numerous studies have reported a relationship between hyperglycemia and brain dysfunction, particularly depression like-behavior and memory loss, in patients with diabetes [52–54]. In this context, data suggest that MGO, a byproduct of glycolysis, is responsible for brain damage, and there are many arguments regarding whether a high-carbohydrate diet causes depression like-behavior or induces depression. The gut microbiome generates different metabolites, and MGO is one of them [55]. Thus, gut microbiome-derived metabolites, MGO functions, and their links to brain depression like-behavior and memory loss have not been thoroughly studied. MGO is regarded as a toxic metabolite because not only does MGO itself increase oxidative stress, but AGEs also have the potential to increase oxidative stress. Since MGO is abundant in the body in hyperglycemic conditions and produced by the intestinal gut microbiota, we hypothesized that MGO might be linked to tryptophan deficits and increased oxidative stress and inflammation, leading to depression like-behavior and memory impairment in the brain. Interestingly, in previous studies, Stoy et al. reported the relationship between Trp metabolism and oxidative stress in patients with Huntington’s disease [56].

In this study, we administered MGO to mice *via* the rectal route to identify its role in the brain through the gut system. We found that as the MGO dose increased, there was a reduction, with no reduction in body weight, in the time that the mice spent in the central area in the OFT test, and the baseline immobility time of mice increased with MGO treatment in the TST and FST (Fig. 1B and D). Furthermore, MGO treated mice shows loss of the ability to feel pleasure, critically decreased the sucrose consumption, and the total distance and spent time in opened arm significantly decreased in EPM test, as a gold standard test for measuring anxiety (Fig. 1E and F). Our NORT for spatial memory demonstrated an impaired recognition percentage in the MGO mice. The arm entry ability of the mice was not hampered in the Y-maze test. Nonetheless, MGO alleviated the spontaneous alterations in mice (Fig. 1H and I). Furthermore, the latency time to reach the target in the BM test significantly increased in comparison to that of CON group (Fig. 1G). Thus, the results of depression like-behavior and working memory deficit tests suggested the ability of MGO to induce brain dysfunction. A study conducted on a distinct part of cognitive function in patients with depression revealed a greater loss of hippocampal activity [57]. Therefore, we investigated whether the effects of MGO on brain dysfunction were mediated by hippocampal damage. Consistent with previous findings, our results indicated that MGO sharply reduced the number of cells in the DG, CA3, and CA1 areas of the hippocampus in brain (Fig. 2A). Moreover, LTP activity dramatically declined upon MGO treatment, as it was induced by neurotransmitter release (Fig. 2B), supporting the pathological changes in the hippocampus of mouse brains.

Depression increases cortisol levels in the hippocampus, impeding the development of neurons in the brain. Depression is a complicated neurological disorder with several targets, and molecular signaling pathways are implicated [58]. MGO induces cognitive impairment and presenilin-1 expression in aged mice [25]. Recently, de Almeida et al., reported the impact of MGO on behavior-related disorders; detailed mechanistic studies have been conducted on why cognitive decline occurs in depression models induced by MGO [59]. The depression-related genes and MGO target genes were intersected to obtain common genes. The KEGG pathway analysis of common genes between depression like-behavior and MGO revealed the pathways mainly involved in synaptic signaling, serotonergic and dopaminergic synapses, cAMP signaling pathways, the PI3K-Akt signaling pathway, and the MAPK signaling pathway, among others. BDNF, IL-6, and IL-10 were identified as hub genes (Supplementary Fig. S1).

In this study, we analyzed neurotransmitter levels and related factors in mice to clarify the underlying mechanisms (Fig. 3A – F). Trp is the main substrate for 5-HT synthesis, and 5-HT continuously plays a role in the brain regarding depressive behavior and memory learning responses [60]. Therefore, we analyzed the effects of MGO on Trp and its derivatives, for instance, 5-HTP and 5-HT, and data from our study showed decreased levels of Trp, 5-HTP, and 5-HT in mouse plasma (Fig. 3A – C). We suggest that the low availability of this enzyme in the human brain is strongly related to depression-like behaviors and memory loss. In this context, we observed the effects of MGO treatment on TPH2 through the histochemical analysis of brain sections. This assessment showed that MGO significantly reduced TPH2 levels in the CA1, CA3, and cortical regions (Fig. 3G), suggesting a further reduction of 5-HT levels in the brain. It is known that 5-HT, DA, and EPI are the representative neurotransmitters involved in depression, working memory, and other cognitive impairments [61, 62]. In our experimental model, MGO displayed remarkable effects in decreasing the levels of DA, EPI, and 5-HT in the mouse brain (Fig. 4D – F). Too little DA, EPI, or 5-HT can impair communication between neurons. Low dopamine levels can cause depression-related symptoms. Therefore, we investigated the effects of MGO on these intestinal factors. Surprisingly, we found that MGO decreased TPH1 levels in the colon tissue, as TPH-1 is mainly expressed in the colon and colon length (Supplementary Fig. S4), indicating a direct gut-brain relationship.

Based on the above results, we hypothesized that MGO might have direct crosstalk with Trp in depressive like-behavior and memory deficits in mice. To test our hypothesis, we initially performed an MGO affinity assay to examine whether MGO has a high affinity for Trp or other amino acids (Supplementary Fig. S5). Surprisingly, MGO showed the highest affinity and trapping ability for Trp compared to other amino acids. In agreement with this result, we treated primary hippocampal neurons without Trp and with or without a combination of MGO and tryptophan (MGO + Trp). After treatment, we observed reduced cell viability and neuronal growth in primary hippocampal cells (Fig. 4C). Here, MGO treatment sharply lowered Trp levels in the cells. We confirmed that this affinity of MGO to Trp is responsible for neuronal cell death in other neuronal cells, whereas treatment without Trp or MGO + Trp decreased neurite growth and the neurite branch point of neuron cells (Fig. 4E). Of specific relevance, no Trp or MGO + Trp diminished the levels of TPH1 and TPH-2 more prominently than treatment with Trp alone in neuronal cells. These results strongly suggest that MGO-triggered depression and memory impairment may be caused by the depletion of Trp and related neurotransmitters.

Tau phosphorylation plays a crucial role in Aβ-induced dendritic or synaptic dysfunction in depression like-behavior and memory decline. The Aβ levels are generated through sequential cleavage of APP [63]. MGO strongly elevated Tau phosphorylation and increased the oAβ and APP levels (Fig. 5A). In addition, NGF and PSD95 expression levels in the brain were dramatically reduced by MGO treatment. It is well established that the BDNF/TrkB-PI3K/Akt/GSK-3β signaling pathway activates Tau phosphorylation and APP [30]. In this study, we assessed the expression levels of these signaling proteins to extend our understanding of their molecular mechanisms. Surprisingly, MGO suppressed BDNF/TrkB protein expression but boosted Akt and GSK-3β phosphorylation. Activated Akt at ser473 inhibits GSK-3β function in tau phosphorylation (Fig. 5A). The possible explanation for this disparity is that MGO might sulfhydrated Akt, which does not interact with GSK-3β [64]. However, MGO was attributed to the reduction of activated GSK-3β (Ser9), suggesting its role in Tau phosphorylation and Aβ deposition (Fig. 5A). Interestingly, Receptor for AGE (RAGE) levels did not change (Fig. 5A). Thus, these findings indicate that other signaling pathways might be involved in MGO-activated Tau phosphorylation and increased Aβ oligomer and APP levels in the brain.

One recent study reported that oxidative stress happened before Aβ plaque formation and Tau phosphorylation in the brain [65]. Disproportionate levels of pro-and antioxidants characterize OS. It is believed that elevated oxidative stress plays a crucial role in the pathogenesis of Aβ plaque formation and Tau phosphorylation [66]. MGO is known to accelerate OS, and the MAPK signaling pathway is activated in oxidative stress. Our study demonstrated that MGO persistently increased the phosphorylation of ERK1/2, JNK, and p-38. ERK1/2 and p-38 phosphorylation might be linked to raise Tau phosphorylation and Aβ formation. MAPK activation, particularly NF-κB, can also cause inflammation [67]. Later, we examined the MGO treatment effects on inflammatory markers and found that MGO remarkably enhanced Iba-1, a microglial activation marker, expression, and phosphorylation of IκB and NF-κB, IL-6, and TNF-α secretion but reduced IL-10 secretion. Our data shows that MGO, a representative RCSs, and ROS synergistically induce severe inflammation simultaneously (Fig. 6). Indeed, we found that MGO treatment activated the microglia in both the hippocampus and cortex area in mice, supporting that MGO induced the neuroinflammation (Fig. 10E).

Finally, we investigated whether the MAPK activation and inflammation induced by MGO occurred through oxidative stress stimulation. The thioredoxin (Trx) and GSH systems are two major antioxidant defense systems in mammalian cells [68]. The Trx system consists of thioredoxin reductase 1 (TXNRD1), thioredoxin (Trx), peroxiredoxin (Prx) like TrxR1, and nicotinamide adenine dinucleotide phosphate (NADPH). The GSH system consists of GSH, NADPH, glutathione reductase, glutaredoxin, and glutathione peroxidase (GPx). To eliminate ROS, these two systems use NADPH as an electron donor and transfer electrons to the substrate *via* several components [69]. In the oxidative stress state, cells synthesize antioxidant defense factors such as Nrf2/HO-1/2 signaling and enzymes such as thioredoxin reductase 1 (TXNRD1), Trx, GSH, and CAT. Our data showed that MGO markedly abolished Nrf2, HO-1, TXNRD1, Trx, CAT, and NADPH activities in mice, suggesting MGO-induced higher oxidative stress levels in mice (Fig. 7A). Some studies have demonstrated that GR levels are upregulated upon stress elevation by targeting BDNF levels [70], and the knockdown of GR ameliorates depression like-behavior in mice [71]. Therefore, increased GR levels play a role in accelerating oxidative stress in the brain. Herein, MGO increased GR expression in mice (Fig. 7A).

Because MGO reduces NADPH activity in mice, the role of MGO in regulating Sirt family proteins remains unclear. Sirts are a family of NADPH-dependent histone deacetylases that play important roles in maintaining the redox status of mammalian cells [51]. To clarify the relationship between MGO and OS, we investigated Sirt protein expression in the brains of MGO-treated mice. Our experimental results indicated that MGO significantly decreased the expression of Sirt-3 and Sirt-5 proteins but did not affect the expression of other Sirt family proteins (Sirt-1, Sirt-2, Sirt-4, and Sirt-7) (Fig. 7E). Moreover, MGO showed great affinity for Sirt-3 and Sirt-5 proteins exhibited by docking analysis. Sirt-3 and Sirt-5 are mitochondrial Sirts that are known to control redox signaling or the production of ROS generated through mitochondrial dysfunction by enhancing SOD1, SOD2, and CAT activity, thus resulting in lower levels of SOD1, SOD2, and CAT activity in the brains of MGO-treated mice (Fig. 7D). These findings suggested that MGO augmented oxidative stress in mice with depression like-behavior and memory dysfunction.

To clarify our hypothesis that MGO-mediated depression like-behavior and impaired memory in mice are due to diminished Trp levels or induction of oxidative stress, we treated depressive mice with Trp, and the outcomes were excluded. In the OFT, Trp-administered mice showed a marked increase in line crossings and time spent in the center (Fig. 8B). Similarly, the immobility time was reduced in Trp-treated mice in the TST and FST (Fig. 8C, D). Also, Trp recovered the depression like-behaviors in the SPT and EPM tests (Fig. 9B, C). Interestingly, Trp increased recognition and arm alteration percentages in mice with MGO-induced depression (Fig. 8E and C, and 9D). Moreover, phosphorylation of Tau, ERK1/2, JNK, and NF-κB was markedly diminished by Trp (Fig. 10D). Likewise, it reduces APP and GR levels, IL-6, and TNF-α secretion (Fig. 10). Trp accelerated MGO detoxification factors, such as GLO I and GLO II, and moderated CAT activity, indicating that the Trp connection to MGO may promote tryptophan antidepression effects (Fig. 10D). In the intestine, Trp dramatically increased colon length in mice, which was reduced by MGO treatment.

**Fig. 11:**
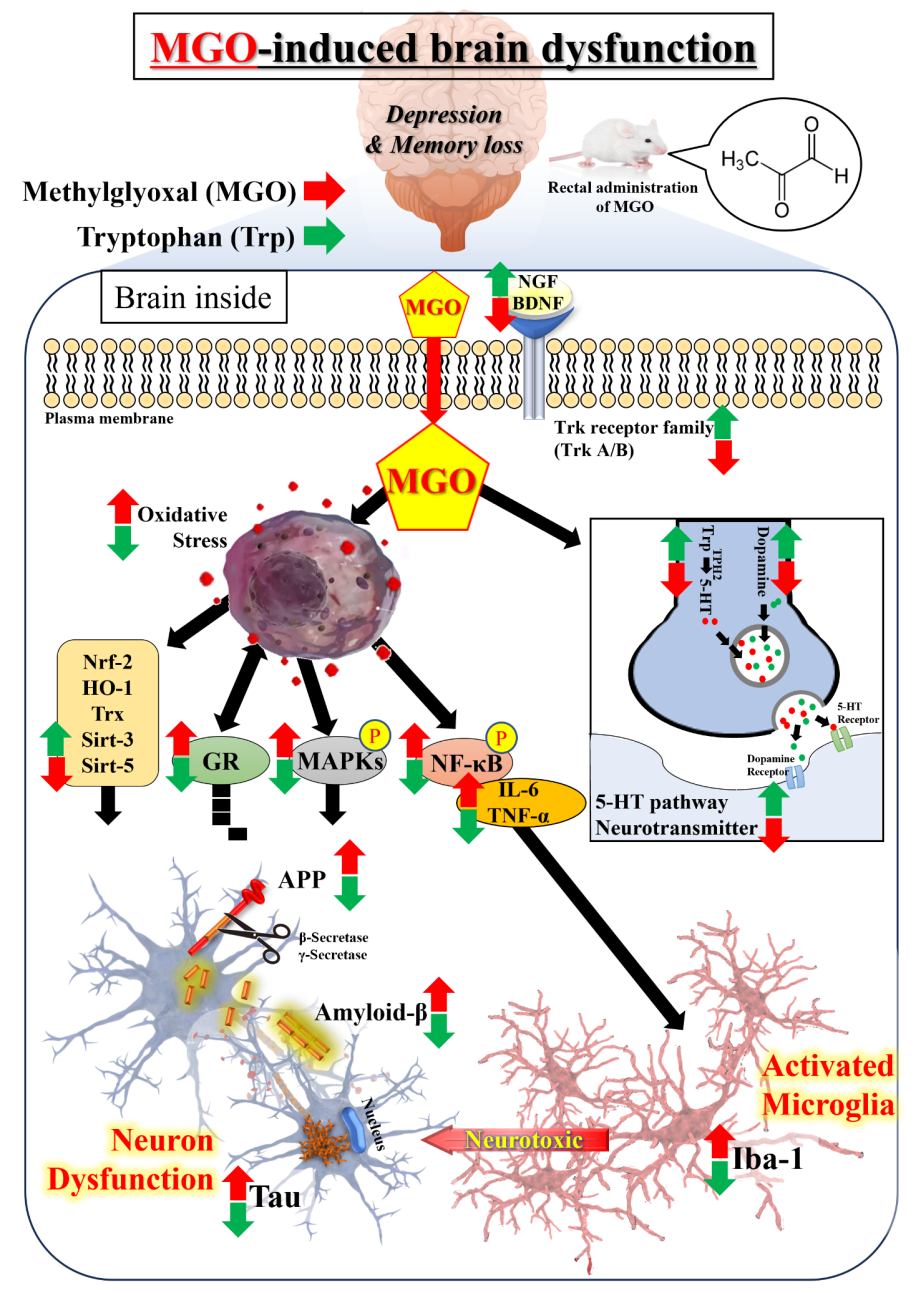
Schematic diagram of the mechanism of methylglyoxal (MGO)-induced depression and memory deficits* via *reducing tryptophan levels and increasing oxidative stress in mice. The mechanism involves the reduction of tryptophan, and neurotransmitters, promotion of Tau, APP and MAPK phosphorylation, enhanced inflammation, and increased oxidative stress *via* downregulating Nrf-2/HO-1, Trx, and Sirt-3/5 antioxidant system

Further research will be required to clarify the casual model proposed in Fig. 11. For example, does MGO suppress the shikimate pathway in the murine gut microbiome, thereby leading to a decrease in the aromatic amino acids [e.g., Trp, tyrosine (Tyr), phenylalanine (Phe)] produced by the gut [72]. If so, this will have consequences for the levels of a number of precursors necessary for the synthesis of neurotransmitters, including DA, and 5-HT. A decrease Trp levels in gut can increase gut permeability *via* the conversion of Trp by tryptophan decarboxylase (TDC) to tryptamine, which activates the aryl hydrocarbon receptor to maintain gut integrity [73]. An increase in gut permeability will have a number of consequences, including the increasing levels of circulating lipopolysaccharide (LPS), which can activate TRL4 on astrocytes and neurons to increase NF-κB and YY1 transcription factors, thereby increasing β-site amyloid precursor protein cleaving enzyme-1 (BACE1) and Aβ production [74]. Increased gut permeability also induces gut dysbiosis, leading to alterations in gut microbiome products including decreased butyrate production. Increased gut permeability and gut dysbiosis and decreased butyrate production. Increased gut permeability is evident in diabetes, suggesting that MGO mediates changes in the gut that are relevant to its systemic and central nervus system (CNS) effects [75]. We are planning to further advanced study such as metabolomics to ascertain whether gut dysbiosis induced by MGO can alter the shikimate pathway.

Although decreased 5-HT is classically associated with depression, more cutting-edge research suggests that role of 5-HT as a precursor for the melatonergic pathway will compromise the capacity of mitochondrial function. A suppressed capacity of mitochondria to induce the melatonergic pathway will comprise the capacity of CNS cells to resist challenge [74]. A decrease in melatonin, from the pineal gland, glia and/or neurons has significant consequences for mitochondrial function that overlaps would overlap with the loss of gut microbiome-derived butyrate. Both melatonin and butyrate increase mitochondrial Sirt-3 [76, 77], with Sirt-3 deacetylating and disinhibiting the mitochondrial pyruvate dehydrogenase complex (PDC), leading to an increased conversion of pyruvate to acetyl-CoA, with increases ATP production by the tricarboxylic acid cycle and oxidative phosphorylation [78].

Sirt-3 also inhibits the levels of oxidants produced by the electron transport chain, indicating that suppression of melatonin and butyrate by MGO will suppress mitochondrial function and increase oxidant production. The suppression of melatonin and butyrate will also increase the levels and effects of corticosteroids and GR activation. Melatonin directly suppresses corticosteroid/cortisol production by the adrenal cortex [79], whilst melatonin and butyrate prevent the activated GR from being translocated to the nucleus [80]. This would therefore indicate that any MGO suppression of melatonin and butyrate will potentiate GR activation and therefore potentiate the processes underpinning diabetes and neurodegeneration.

Overall, the investigation of such processes would indicate tat alteration in core aspects of cell function, namely mitochondrial metabolism and oxidant production, would be casually upstream on the changes in intracellular signaling pathways highlighted herein. The investigation of these processes in future research should better clarify the causal modeling underpinning how MGO associated with diabetes, mood disorders and neurodegenerative conditions.

## Conclusions

In summary, we report for the first-time exclusive insights into the mechanism of rectal administration of MGO-induced mice model. MGO sharply reduced Trp and neurotransmitter (i.e., DA, EPI, 5-HT) levels and their metabolism-related factors, TPH1 and TPH2, and hippocampal cells in the brain. Interestingly. Furthermore, treatment with MGO+Trp displayed effects similar to those exhibited by Trp (-)-treatment in neuronal cells, such as the induction of cell toxicity, decreased TPH1 and TPH2 levels, dendritic spine density, and neuronal outgrowth. Importantly, MGO enhanced the Phosphorylation of Tau and GSK-3β and downregulated BDNF/TrkB or NGF signaling pathways. Moreover, it activates MAPK (ERK1/2) signaling by increasing oxidative stress, which suppresses Nrf-2, HO-1, TXNRD1, Trx, CAT, Sirt-3, and Sirt-5 antioxidant components. Additionally, MGO elevated inflammation by activating NF-κB and increasing IL-6 and TNF-α secretion. Notably, Trp treatment reversed the effects of MGO in the mouse brain. Here, we report a novel in vivo model of MGO-induced depression and memory loss induced by MGO through Trp depletion, inflammation, and oxidative stress (see Fig. 11).

## Electronic supplementary material

Below is the link to the electronic supplementary material.

Supplementary Material 1

## Data Availability

This study includes no data deposited in external repositories. All data are included in this article and supplementary files are available upon request from the corresponding author.

## Abbreviations

DM: Diabetes mellitus
MGO: Methylglyoxal
TPH1: Tryptophan hydroxylase 1
TPH2: Tryptophan hydroxylase 2
5-HT: 5-hydroxytryptamine
DA: Dopamine
EPI: Epinephrine
DG: Dentate gyrus
CA3: Cornu ammonis 3
CA1: Cornu ammonis 3
CAT: Catalase
Nrf-2: Nuclear factor erythroid-related factor-2
Ho-1: Heme Oxygenase-1
Sirt: Sirtuins
NAD: Nicotinamide adenine dinucleotide
TXNRD1: Thioredoxin reductase-1
GLO-I: Glyoxalase-I
GLO-II: Glyoxalase-II
Trp: Tryptophan
