# Supplementary material for: Depression like-behavior and memory loss induced by methylglyoxal is associated with tryptophan depletion and oxidative stress: a new in vivo model of neurodegeneration
Source: Biol Res. 2024 Nov 21;57:87. doi: 10.1186/s40659-024-00572-4 (PMC11580208; doi:10.1186/s40659-024-00572-4)
Supplement: Supplementary file 1 — Supplementary Material 1 [file 40659_2024_572_MOESM1_ESM.docx]

**<Supplementary data>**

**Title: Depression like-behavior and memory loss induced by methylglyoxal is associated with tryptophan depletion and oxidative stress: a new *in vivo* model of neurodegeneration**

**<Supplementary methods>**

**Bio-informatic analysis**

Depression-associated targets were obtained from the GeneCards database, and potential targets of MGO were retrieved from PALM-IST and validated using the PubMed database. Common genes were identified by intersecting MGO and depression targets using Vinny 2.1. First, gene ontology (GO) and KEGG pathway analysis were performed using the ShinyGO online tool. The common genes were then uploaded to the STRING database for the protein-protein interaction network, and the file was saved to obtain the hub gene. Hub genes were recognized using the Cytoscape software.

**MGO-affinity assay**

The MGO-affinity assay was performed to analyze the reaction at different stages of the MGO-amino acid compound process, according to the method of Ina Nemet *et. al.* with slight modifications [1]. Briefly, MGO in the presence or absence of amino acids was incubated in PBS (pH 7.4) and 0.02% sodium azide for 7 d at 37°C in the dark. The affinity of MGO-amino acid was evaluated in terms of fluorescence at excitation/emission wavelengths of 355/460 nm, as detected using a VICTOR™ X3 multilabel plate reader (PerkinElmer, MA, USA).

**Histological analysis**

For immunohistochemical analysis, brain and colon sections were incubated in 0.1% protease K in phosphate-buffered saline (PBS) for antigen retrieval and incubated in 3% H_2_O_2_ in PBS for 15 min. Sections were incubated with 1% normal horse serum in phosphate-buffered saline (PBS) for 20 min. After blocking, the sections were incubated with TPH1 (1 : 100, Abcam, UK) and TPH2 (1 : 100, Abcam, UK) over night in a shaker at 4°C. The sections were washed and incubated with biotinylated respective IgG secondary antibodies at room temperature (25°C) for 1 h. The sections were then rinsed with PBS and incubated with VECTASTAIN® ABC reagent for 30 min. The antibody expression was detected using 3,3-diaminobenzidine (DAB). The stained tissue slides were observed and photographed using a Nikon Eclipse 80i microscope (Nikon, Tokyo, Japan) at 100× magnification, and detected intensity was calculated using the ImageJ software (NIH, Bethesda, MD).

**<Supplementary figure legends>**

**Fig. S1. Bioinformatics analysis of MGO and depression-related target genes.** (A) The Venn diagram approach was used to identify the common targets between MGO and depression. (B, C) Go Biological process and KEGG pathway analysis of common targets of two groups. (D) Finding hub genes from intersected genes using Cytoscape software

**Fig. S2.** **Immunostaining analysis of brain sections was performed to examine TPH1 levels in the cortex region.** Stained images represent TPH1 levels in the cortex regions indicated in the box. Scale bar: 50 – 100 µm. Quantitative measurements of TPH1 intensity were conducted by calculating the expression in the cortex from selected fields per image. The values were calculated using ImageJ software. The values are presented as mean ± SEM (n = 3) ^#^*p* < 0.05 *vs.* control group (CON).

**Fig. S3: MGO modulated colon length and tryptophan metabolism in mice colon tissue.** (A) The colon was measured using a ruler (in cm). (B) Representative images were stained for TPH1 and TPH2 in the colon tissues. Scale bar: 50 µm. Quantitative measurements of TPH1 and TPH2 were conducted by calculating the expression in the colon from selected fields per image. The values were calculated using ImageJ software. The values are presented as mean ± SEM (n = 3 – 8). ^#^*p* < 0.05 and ^##^*p* < 0.01 *vs.* control group (CON).

**Fig. S4: Trp recovered colon length in MGO-induced mice model.** The normal control group received only 30% v/v glycerol in PBS, while the MGO group received MGO diluted in PBS, and the Trp group was given MGO and Trp. After sacrifice, the colon was measured using a ruler (in cm). The values are presented as mean ± SEM (n = 8). ^#^*p* < 0.05 *vs.* control group (CON).

**Fig. S5: Affinity of MGO towards amino acids and trapping-ability towards Trp.** An affinity assay was performed to analyze the reaction between MGO and amino acids. The amino acids (1 mM) were incubated with MGO (10 mM) for 7 days. The affinity value of the MGO amino acid complex was evaluated using fluorescence at excitation/emission wavelengths of 355/460 nm. The values are presented as mean ± SEM (n = 3). ^#^*p* < 0.05 vs. PBS. ^***^*p* < 0.001 *vs.* normal (PBS+MGO 10 mM).

**Reference**

1. Nemet I, Varga-Defterdarovic L. The role of methylglyoxal in the non-enzymatic conversion of tryptophan, its methyl ester and tryptamine to 1-acetyl-beta-carbolines. Bioorg Med Chem 2008; 16(8):4551-4562.

**Fig. S1.**

**
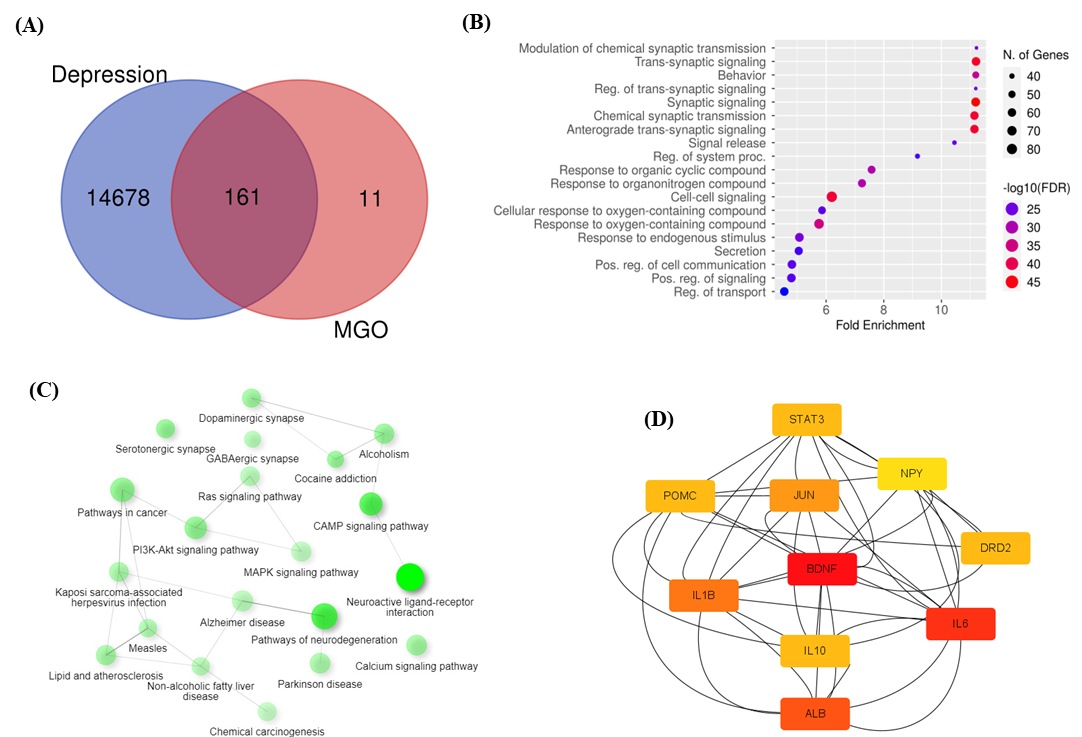
**

**Fig. S2.**


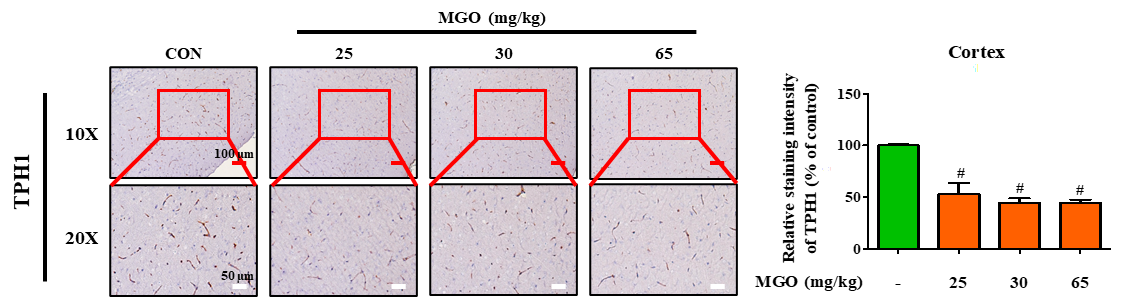


**Fig. S3.**


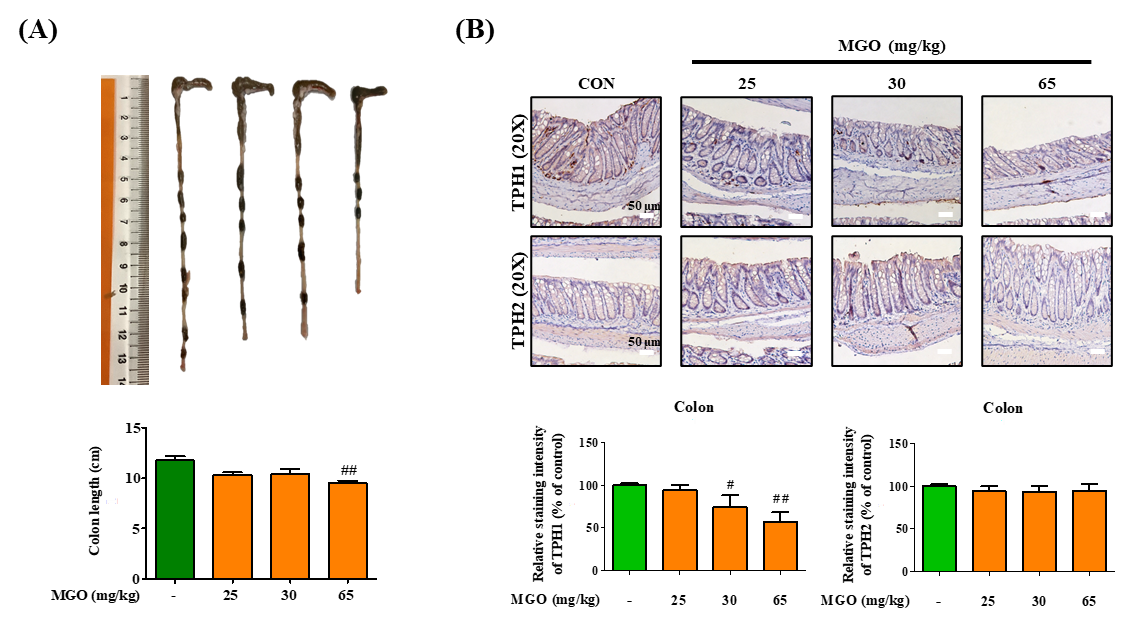


**Fig. S4.**


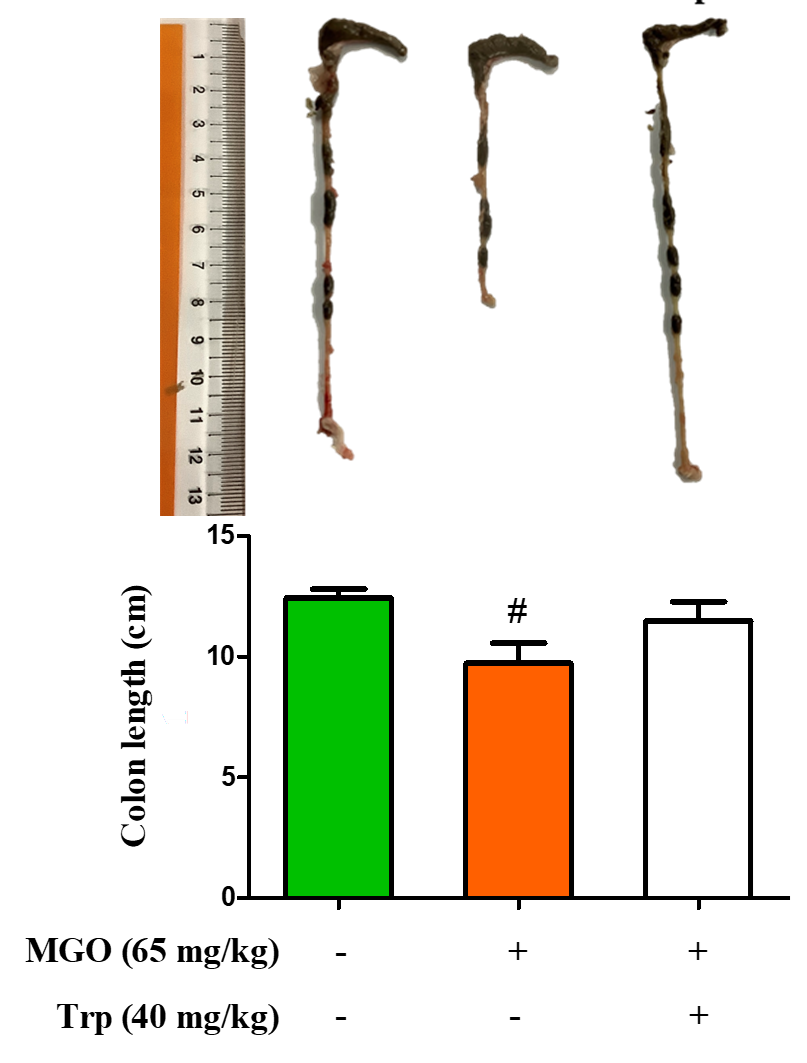


**Fig. S5.**

**Table S1. Lists of primers used in qRT-PCR analysis.**

| Gene | Direction | Sequence (5’ to 3’) |
| --- | --- | --- |
| TPH1 | Forward | TTCACCATGATTGAAGACAAC |
|  | Reverse | TCCGACTTCATTCTCCAAGG |
| TPH2 | Forward | CCATCGGAGAATTGAAGCAT |
|  | Reverse | TTCAATGCTCTGCGTGTAGG |
